# ANGPTL8 has both endocrine and autocrine effects on substrate utilization

**DOI:** 10.1172/jci.insight.138777

**Published:** 2020-09-03

**Authors:** Federico Oldoni, Haili Cheng, Serena Banfi, Viktoria Gusarova, Jonathan C. Cohen, Helen H. Hobbs

**Affiliations:** 1 Departments of Molecular Genetics and Internal Medicine, University of Texas Southwestern Medical Center, Dallas, Texas, USA.; 2 Regeneron Pharmaceuticals, Tarrytown, New York, USA.; 3 Center for Human Nutrition, and; 4 Howard Hughes Medical Institute, University of Texas Southwestern (UTSW) Medical Center, Dallas, Texas, USA.

**Keywords:** Endocrinology, Metabolism, Adipose tissue, Lipoproteins, Mouse models

## Abstract

The angiopoietin-like protein ANGPTL8 (A8) is one of 3 ANGPTLs (A8, A3, A4) that coordinate changes in triglyceride (TG) delivery to tissues by inhibiting lipoprotein lipase (LPL), an enzyme that hydrolyzes TG. Previously we showed that A8, which is expressed in liver and adipose tissue, is required to redirect dietary TG from oxidative to storage tissues following food intake. Here we show that A8 from liver and adipose tissue have different roles in this process. Mice lacking hepatic A8 have no circulating A8, high intravascular LPL activity, low plasma TG levels, and evidence of decreased delivery of dietary lipids to adipose tissue. In contrast, mice lacking A8 in adipose tissue have higher postprandial TG levels and similar intravascular LPL activity and plasma A8 levels and higher levels of plasma TG. Expression of A8, together with A4, in cultured cells reduced A4 secretion and A4-mediated LPL inhibition. Thus, hepatic A8 (with A3) acts in an endocrine fashion to inhibit intravascular LPL in oxidative tissues, whereas A8 in adipose tissue enhances LPL activity by autocrine/paracrine inhibition of A4. These combined actions of A8 ensure that TG stores are rapidly replenished and sufficient energy is available until the next meal.

## Abstract 2

Angiopoietin-like protein ANGPTL8 expressed in liver and adipose tissue partner with ANGPTL3 and ANGPTL4 respectively, and replenish adipose tissue triglyceride stores by distinct endocrine and autocrine/paracrine mechanisms.

## Graphical abstract

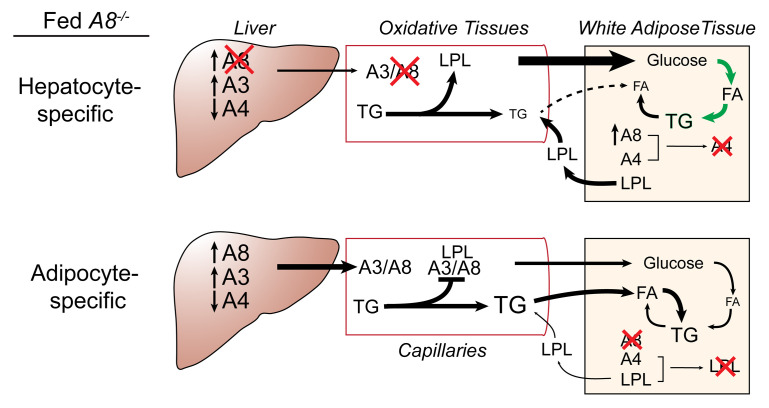

## Introduction

Fatty acids from the diet or synthesized de novo are esterified with glycerol to form triglycerides (TGs), the major energy reservoir in vertebrates. In enterocytes and hepatocytes, TGs are packaged with other lipids and proteins and secreted into the circulation as components of chylomicrons or VLDL (1, 2). Lipoprotein lipase (LPL), an enzyme residing on the endothelial surfaces of capillaries, hydrolyzes circulating TGs, releasing fatty acids for uptake by adjacent tissues (3, 4). The fraction of TG-derived fatty acids delivered to each tissue is determined by LPL activity in that tissue (5). In fasted animals, fatty acids are delivered predominantly to oxidative tissues (muscle, heart, brown fat) where LPL activity is high (5, 6). In the fed state, LPL activity is suppressed in oxidative tissues so that more fatty acids traffic to white adipose tissue (WAT) (5, 6). Uptake of fatty acids into WAT is 10-fold higher in fed mice than in fasting animals (7).

The rapid changes in LPL activity in tissues in response to nutritional intake are mediated primarily at the posttranslational level (8) by 3 members of the angiopoietin-like protein (ANGPTL) family of secreted proteins: ANGPTL3 (A3), ANGPTL4 (A4), and ANGPTL8 (A8) (9–11). All 3 proteins inhibit LPL in vitro, albeit with different potencies (12–14). The ANGPTLs are expressed in a tissue-specific manner, and levels of expression depend on nutritional cues (7, 15, 16). All 3 proteins are highly expressed in liver. Whereas A3 expression is restricted almost exclusively to liver (9, 17), A4 and A8 are also expressed at high levels in adipose tissue (11, 15, 18–20). Feeding elicits dramatic and reciprocal changes in A4 and A8 mRNA levels. Levels of A4 mRNA increase in WAT during fasting and fall upon refeeding (15), whereas expression of A8 is barely detectable in the tissues of fasted animals but increases rapidly upon refeeding (11, 19, 20). Levels of A3 mRNA in the liver are consistently higher than those of either A4 or A8 and change more modestly in response to food intake (21).

A4 and A3 have similar overall structures. Both contain a proprotein convertase site and can undergo catalytic cleavage, producing an N-terminal coiled-coil domain that contains an LPL interaction site, and a C-terminal fibrinogen-like domain (22). A8, unlike A3 or A4, does not contain a fibrinogen-like domain. A4 is a more potent inhibitor of LPL in vitro and in vivo than the other 2 family members (13, 14) and is essential for the fasting-induced suppression of intravascular lipolysis (15). A4 also stimulates intracellular lipolysis in WAT (23, 24). Thus, A4 expression in WAT promotes flux of fatty acids toward oxidative tissues to supply them with energy substrate during fasting.

A3 and A8 are functionally interdependent in vivo (11) and in vitro (25). Expression of A8 fails to suppress intravascular LPL of mice lacking A3 (11). The 2 family members form a complex in which the LPL binding domain of A8 (and not A3) is required for LPL inhibition (25). After feeding, A3 and A8 act together in the systemic circulation to inhibit LPL activity. Under these conditions, more circulating TGs bypass oxidative tissues and are hydrolyzed in WAT (7, 21). Feeding also increases expression of A8 in adipose tissue, but its role in WAT has not been defined (11, 19, 20).

To begin to understand the relative roles of A8 in liver and in adipose tissue, we developed mice in which A8 is selectively inactivated in those 2 tissues. Here we show that A8 has distinct physiological functions in the 2 tissues. All detectable circulating A8 originates in the liver, and only this form of A8 complexes with A3 to inhibit intravascular lipolysis. A8 from adipose tissue makes no detectable contribution to circulating A8, but rather has local effects on substrate homeostasis. A8 expression in adipose tissue attenuates the LPL inhibitory actions of A4, thus ensuring a rapid replenishment of energy stores with feeding after a fast.

## Results

### Tissue-specific inactivation of A8.

**Figure 1:**
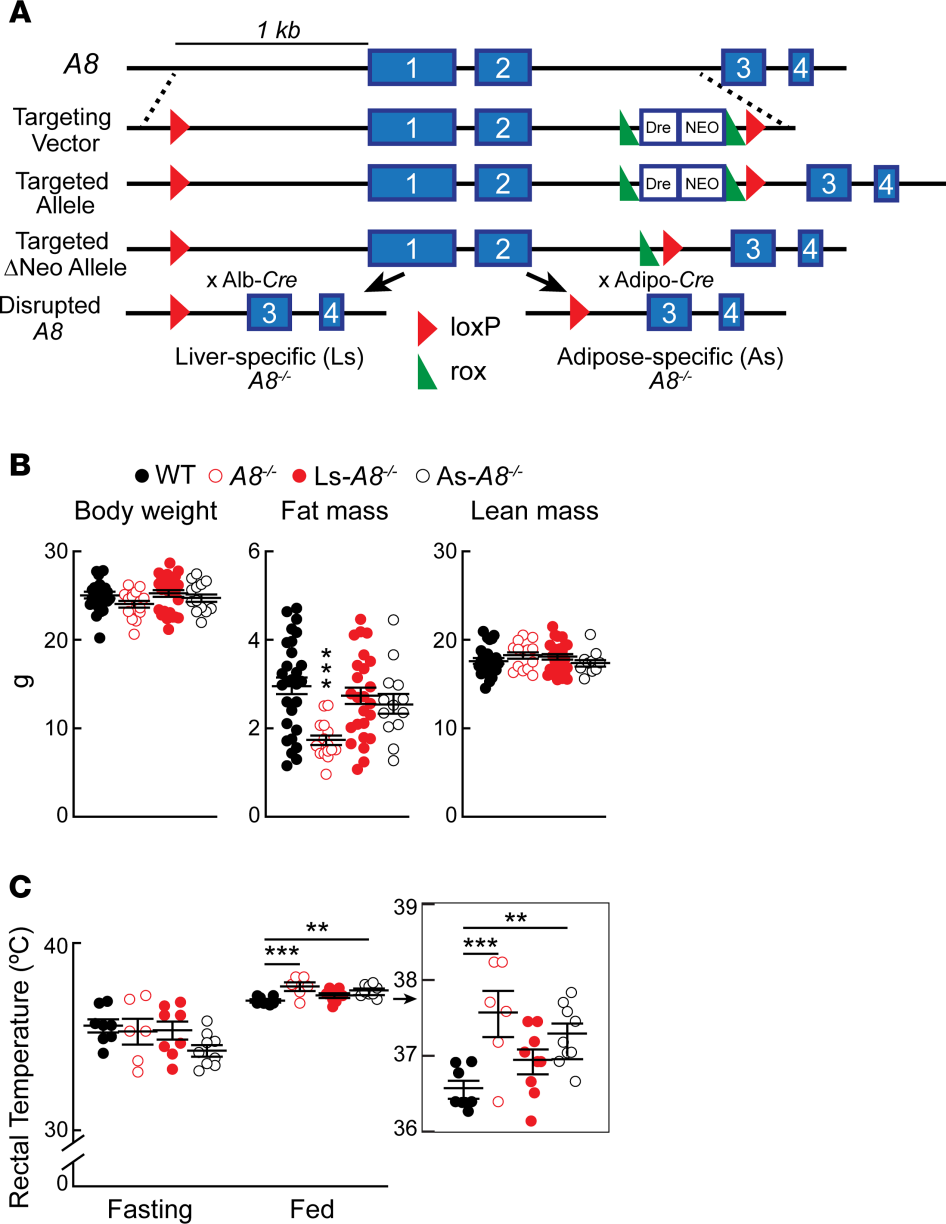
Body weights and rectal temperatures of tissue-specific A8 knockout mice. (**A**) Schematic of mouse *A8*, the targeting vector, the targeted allele, the targeted ΔNeo allele, and the disrupted *A8* allele. *LoxP* sites were placed upstream of exons 1 and in intron 2. A rox-Dre-NEO-rox-loxP cassette was inserted in intron 2. After selection for neomycin (Neo), *Dre* recombinase was expressed to excise the Neo cassette. Mice with the targeted ΔNeo allele (*fl/fl*) were crossed with mice expressing *Cre* under the control of the albumin or adiponectin promoter to inactivate *A8* in hepatocytes (Ls-*A8^–/–^* mice) or in adipocytes (As-*A8^–/–^* mice). (**B**) Mean (± SEM) body weights were determined, and then fat mass and lean body mass of chow-fed male mice (*n* = 13–28/genotype, 9–10 weeks) were measured using nuclear magnetic resonance (NMR). (**C**) Male mice (*n* = 6–9/genotype, 9–10 weeks) were maintained at room temperature (21°C–23°C). Rectal temperatures were obtained at the end of a 15-hour fast (left) and then 4 hours after chow was provided to the mice (right). The experiments were repeated 3 times and the results were similar. Values are shown as means ± SEM. Groups were compared using 1-way ANOVA with Dunnett’s multiple-comparisons test. **P < 0.01; ***P < 0.001.

We developed C57BL/6N mice in which expression of A8 was ablated either in hepatocytes (liver-specific–*A8^–/–^* mice; Ls-*A8^–/–^* mice) or in adipocytes (adipose-specific–*A8^–/–^* mice; As-*A8^–/–^* mice). The mice were established using the *Cre*-lox system to remove exons 1 and 2 of *A8* (Figure 1A). In the targeting construct, the neomycin gene is flanked by 2 *rox* sites that recombine to remove the Neo cassette, thus producing an allele with *loxP* sites flanking the first 2 exons of *A8*. Mice homozygous for the *fl* allele were used as controls for all experiments described in this paper, unless otherwise stated, and are referred to as WT. *A8* was inactivated by crossing mice homozygous for the *fl* allele either with mice expressing *Cre* under control of the albumin promoter (B6N.Cg-*Speer6-ps1^Tg(Alb-cre)21Mgn^*/J; Alb-*Cre*) (26) or with mice expressing *Cre* driven by the adiponectin promoter (Adipo-*Cre*) (27) to produce Ls-*A8^–/–^* mice and As-*A8^–/–^* mice, respectively. Both sexes transmitted the inactivated *A8* allele, and the genotypes of the offspring conformed to the expected Mendelian ratios (Supplemental Table 1; supplemental material available online with this article; https://doi.org/10.1172/jci.insight.138777DS1). Litter sizes were similar in KO and WT mice (Supplemental Table 2). Because A8 is not expressed in the fasting state, all experiments were performed in refed conditions unless otherwise stated.

The mean body weights of the C57BL/6N *A8^–/–^* mice were similar to those of WT controls at 9–10 weeks of age (Figure 1B). The mean total fat mass was significantly lower in the total *A8^–/–^* mice (3.0± 0.2 vs. 1.7± 0.1 g) but not in the tissue-specific KO strains when compared with the WT animals (Figure 1B). Presumably the decreased fat mass in the total *A8^–/–^* mice is compensated by an increase in nonfat mass, although no significant differences in lean body mass were found between the strains. We compared the weights of selected other organs, including the heart, skeletal muscle, spleen, and kidney and found no differences. In a group of older male mice (*n* = 5/group, ages 10–15 weeks, age matched), a small but significant decrease in mean body weight was seen in the *A8^–/–^* mice (25.2 ± 0.3 g vs. 23.0 ± 0.5 g, *P* < 0.04) (Supplemental Figure 1A). These findings are consistent with data from our prior experiments using C57BL/6J *A8^–/–^* mice (28). Also consistent with our former observations, the body weights of the female *A8^–/–^* mice did not differ from the WT mice (Supplemental Figure 1B). The 4 groups of mice were housed in metabolic cages for a week to monitor food intake, activity level, maximal oxygen uptake (VO_2_) uptake, and maximal carbon dioxide uptake (VCO_2_) elimination. No differences in these parameters were seen among the 4 strains (Supplemental Figure 1C). The As-*A8^–/–^* mice consumed slightly more food than the other strains in this experiment, but this difference was not apparent when the experiment was repeated. These results differ from our previous observations in *A8^–/–^* mice on a C57BL/6J background, which had increased VO_2_ in the fed state (28). Genetic differences in fuel homeostasis between C57BL/6J and C57BL/6N have been documented previously (29), which may contribute to the differences in phenotype between the 2 strains of *A8^–/–^* mice.

### Diet-induced hyperthermia in As-A8^–/–^ mice.

Previously, we found that C57BL/6J *A8^–/–^* mice maintained at room temperature (23°C) had higher core body temperatures than WT mice when fed ad libitum but not when fasting (28). Similarly, no temperature differences were found between the 4 strains of mice after a 15-hour fast. However, when measured 4 hours after refeeding, rectal temperatures were significantly higher in the *A8^–/–^* and the As-*A8^–/–^* mice than in the WT animals (Figure 1C). The temperatures of the Ls-*A8^–/–^* mice did not differ from those of WT mice. Consistent with our published results on *A3^–/–^A8^–/–^* mice, no increase in temperature in refed As-*A8^–/–^* mice was observed under conditions of thermoneutrality (Supplemental Figure 1C). We concluded from this experiment that the increase in body temperature in the fed *A8^–/–^* is caused by a lack of A8 expression in adipose tissue.

### Reduced fat pad mass and cell size in WAT of A8^–/–^ mice.

**Figure 2:**
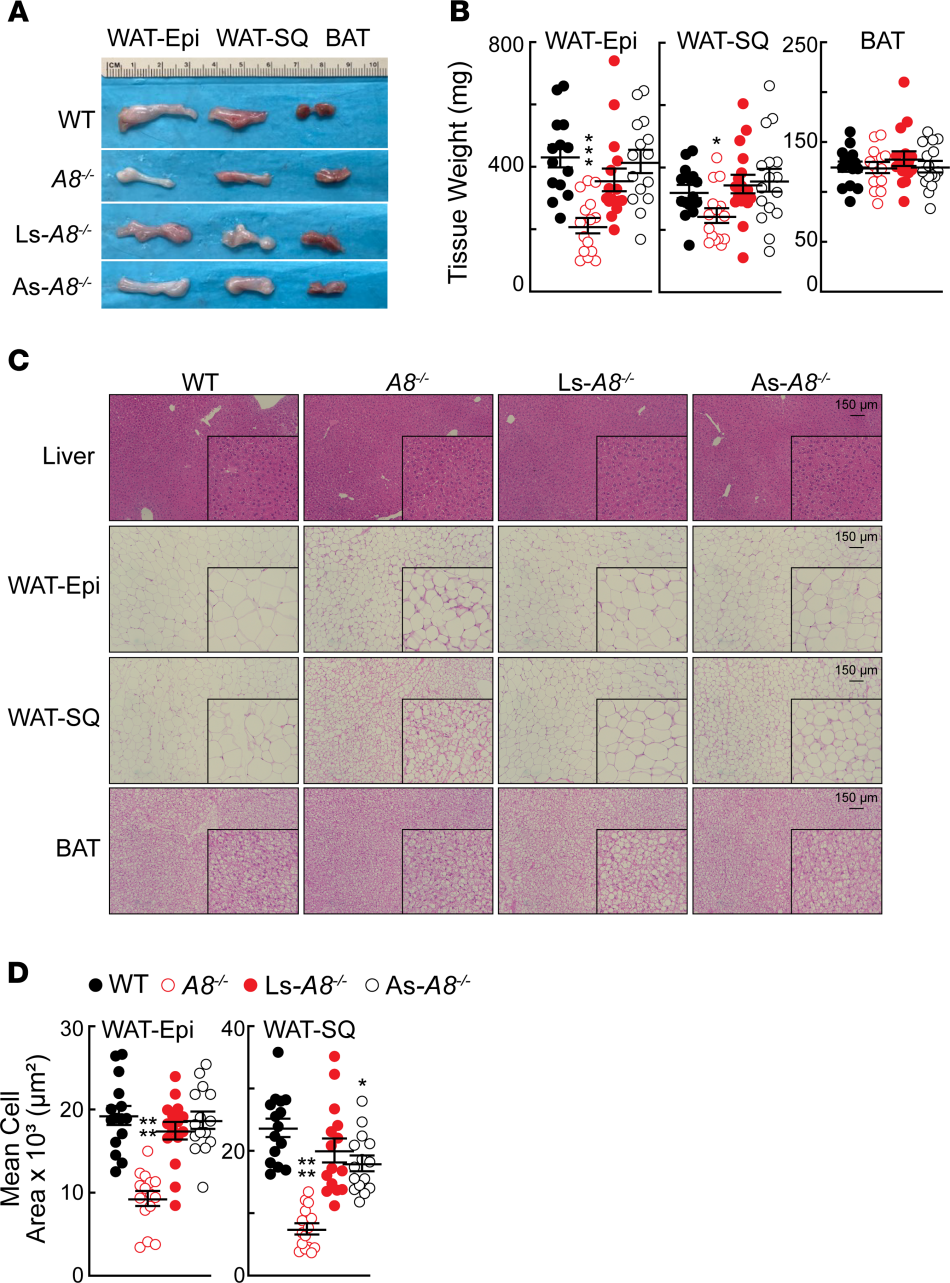
Adipose tissue morphology in chow-fed mice of the indicated genotypes. (**A**) Representative images of WAT and BAT fat pads in age-matched male mice (6/genotype, 11–12 weeks). (**B**) Mean tissue weights (± SEM) of fat pads from age-matched male mice (*n* = 4–5/genotype, 9–14 weeks). Data were pooled from 3 independent experiments (*n* = 14–16/genotype). Values were compared using 2-way ANOVA. (**C**) Representative H&E staining of tissue sections (*n* = 6/genotype; 12–13 weeks). Original magnification, ×20; scale bars: 1 mm = 150 μm). (**D**) Adipocyte size was quantified using ImageJ (NIH): 3 fields/tissue section. Values are shown as means (± SEM) from 3 fields. Groups were compared using 1-way ANOVA with Dunnett’s multiple-comparisons test. *P < 0.05; ***P < 0.001 (**B**); ****P < 0.0001 (**D**). WAT, white adipose tissue; -Epi, epididymal; -SQ, subcutaneous; BAT, brown adipose tissue.

The fat pads were dissected from adjacent tissues and weighed. Representative fat pads from 12-week-old male mice are shown in Figure 2A. The epididymal (Epi) fat pads from the *A8^–/–^* mice were visibly smaller and weighed significantly less than those from WT animals (Figure 2B), which is consistent with what was seen in the *A3^–/–^A8^–/–^* mice (28) and the *A8^–/–^* rat (30). The weights of the WAT-subcutaneous (SQ) fat were also lower in the *A8^–/–^* mice. Microscopic inspection of the fat pads revealed that cell size appeared to be smaller in both the WAT-Epi and -SQ fat from the *A8^–/–^* mice (Figure 2C). On average, adipocytes from both WAT depots were smaller than from the corresponding fat depots of WT controls (Figure 2D). We noted no differences among the strains in the morphology (Figure 2A) or histology (Figure 2C) of the interscapular BAT depots. The observation that the *A8^–/–^* mice, but not the tissue-specific *A8^–/–^* mice, had smaller fat pads suggests that expression of A8 in either liver or WAT is sufficient to maintain normal TG accumulation in WAT.

### Circulating A8 is liver derived.

**Figure 3:**
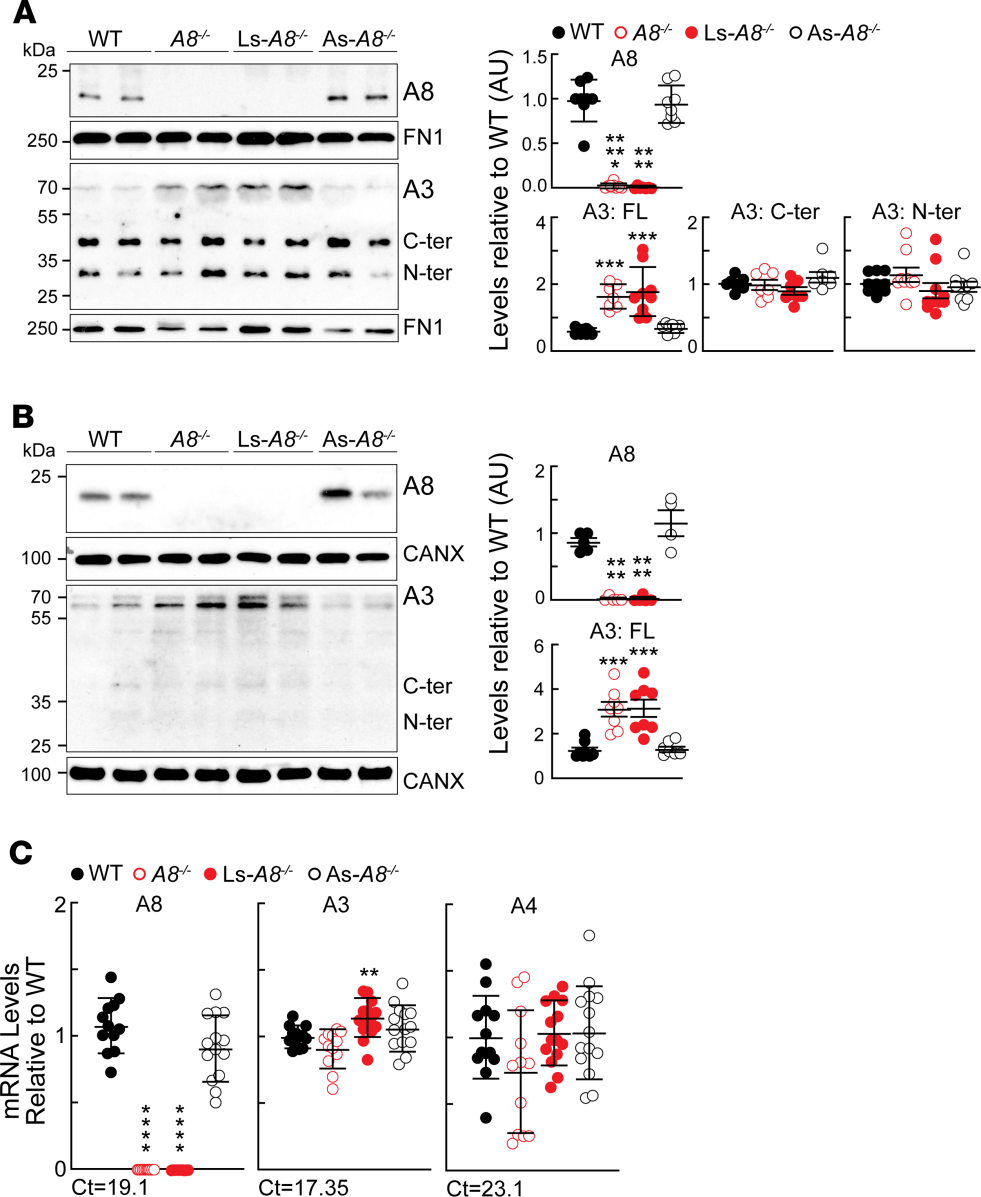
Immunoblot analysis of A3 and A8 in plasma and liver lysates in mice of the indicated genotypes. Age-matched, chow-fed male mice (*n* = 7–8/genotype, 9–12 weeks) were subjected to a 3-day fasting and refeeding protocol, as described in the Methods. All experiments were performed in refed conditions. Immunoblot analysis of (**A**) plasma samples (1.5 μL) and (**B**) liver lysates (50 μg) was performed using anti-A8, anti-A3, and anti-fibronectin (FN1) or anti-calnexin (CANX) antibodies as described in the Methods. The intensity of the bands was determined by LI-COR Image Studio Lite. Data were pooled from 2 experiments (*n* = 3–4/group/experiment). Values represent means (± SEM). Groups were compared using 2-way ANOVA. (**C**) mRNA levels of hepatic A3, A4, and A8 were determined by real-time PCR on 3 independent groups of animals (*n* = 4–5/genotype), and the results were pooled and expressed relative to WT mice as described in the Methods. Cycle threshold (Ct) values in the WT mice are provided. Groups were compared using 2-way ANOVA. **P < 0.01 (**C**); ***P < 0.001 (**A** and **B**); ****P < 0.0001 (**A**, **B**, and **C**); *****P < 0.00001 (**A**).

To determine the relative contributions of liver and WAT to circulating A8, we immunoblotted plasma samples from WT, *A8^–/–^*, Ls-*A8^–/–^*, and As-*A8^–/–^* mice using an anti-A8 monoclonal antibody (Figure 3A). An immunoreactive band migrating at the expected position of A8 (22 kDa) was observed in plasma of WT and As-*A8^–/–^* mice. No A8 protein was detected in the plasma of Ls-*A8^–/–^* or *A8^–/–^* mice. These data indicate that circulating A8 comes predominantly (and likely exclusively) from liver, with virtually no contribution from adipose tissue. It remains possible that some A8 synthesized in adipose tissue enters the circulation but is cleared so rapidly that it avoids detection in our analyses. Similar analysis of A3 in plasma revealed bands corresponding in size to full-length A3 (70 kDa) and the N- and C-terminal fragments of A3 (31 kDa and 38 kDa, respectively). The amount of the full-length protein was higher in the total *A8^–/–^* mice, as we previously observed (21), and in the Ls-*A8^–/–^* mice (Figure 3A). Thus, A8 expression in the liver is associated with a reduction in circulating levels of full-length A3 but does not appear to alter the cleavage of A3.

To determine whether higher levels of circulating A3 in *A8^–/–^* and Ls-*A8^–/–^* mice were due to increased expression of A3 in liver, we performed immunoblot analysis on liver lysates from animals that were perfused with saline before tissue collection. As expected, we observed no A8 in liver lysates from the *A8^–/–^* and Ls-*A8^–/–^* mice (Figure 3B). Two bands of the expected size for the unglycosylated (67 kDa) and fully glycosylated (70 kDa) forms of A3 were seen in all 4 groups of mice. Only faint bands corresponding to the N- and C-terminal fragments of A3 were observed. Expression levels of full-length A3 were higher in livers of *A8^–/–^* and Ls-*A8^–/–^* mice compared with WT or As-*A8^–/–^* mice (Figure 3B). In the Ls-*A8^–/–^* mice, the 3-fold increase in full-length A3 protein was associated with a more modest 1.2-fold increase in A3 mRNA levels (Figure 3C). Thus, the higher levels of A3 protein in liver lysates and plasma of the *A8^–/–^* and Ls-*A8^–/–^* mice do not appear to be a consequence of differences in *A3* transcription. The results are consistent with a model in which A8 either promotes the intracellular degradation of A3 or accelerates clearance of A3 from the circulation. Additional experiments will be required to determine how A8 alters the expression of A3 in the liver and circulation.

### As-A8^–/–^ mice have no immunodetectable A8 in WAT and BAT.

**Figure 4:**
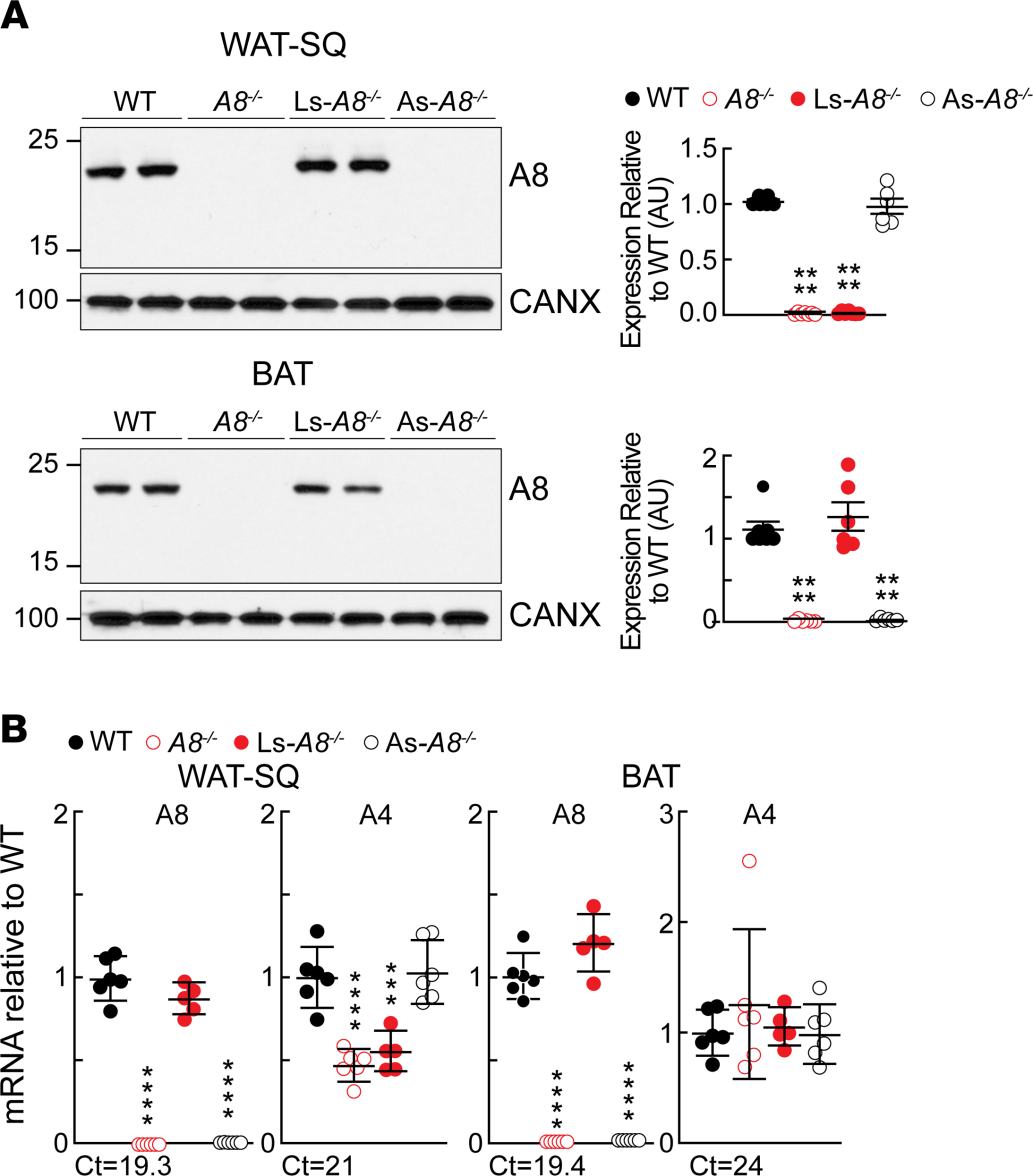
A8 protein and mRNA levels in WAT and BAT of mice of the indicated genotypes. (**A**) Immunoblot analysis of A8 in tissue lysates from WAT-SQ and BAT (25 μg each) of chow-fed male mice (*n* = 3–4/genotype, 12–13 weeks) was performed as described in the Methods. All experiments were performed in refed conditions. Pooled data from 2 experiments (*n* = 3–4/group for each experiment) are shown. Band intensities were normalized to the level of calnexin and then expressed relative to WT. (**B**) mRNA levels of A8 and A4 were determined by real-time PCR in chow-fed male mice (*n* = 6/group, 12–13 weeks). Values are expressed as ratios compared with the mean level in WT littermates, which was set at 1. Ct values in the WT mice are provided. ***P < 0.001; ****P < 0.0001. Groups were compared using 2-way ANOVA. The experiments were repeated twice and the results were similar.

Immunoblot analysis of A8 in lysates from WAT-SQ and BAT in the fed state in the 4 strains of mice is shown in Figure 4A. No A8 was detected in the lysates from *A8^–/–^* and As-*A8^–/–^* mice. While we could not reliably detect A4 in tissues of mice using any of the available antibodies against A4, the levels of A4 mRNA were significantly lower in the WAT-SQ but not in the BAT of the *A8^–/–^* and Ls-*A8^–/–^* mice (Figure 4B). The lower levels of A4 mRNA in the WAT-SQ of these mice likely reflects the decrease in delivery of TGs to WAT (21). A4 has been shown to be regulated by the transcription factor peroxisome proliferator–activated receptor–γ (15), which is responsive to cellular fatty acid levels (31).

### Absence of A8 in liver and adipose tissue has opposite effects on plasma TG levels.

**Figure 5:**
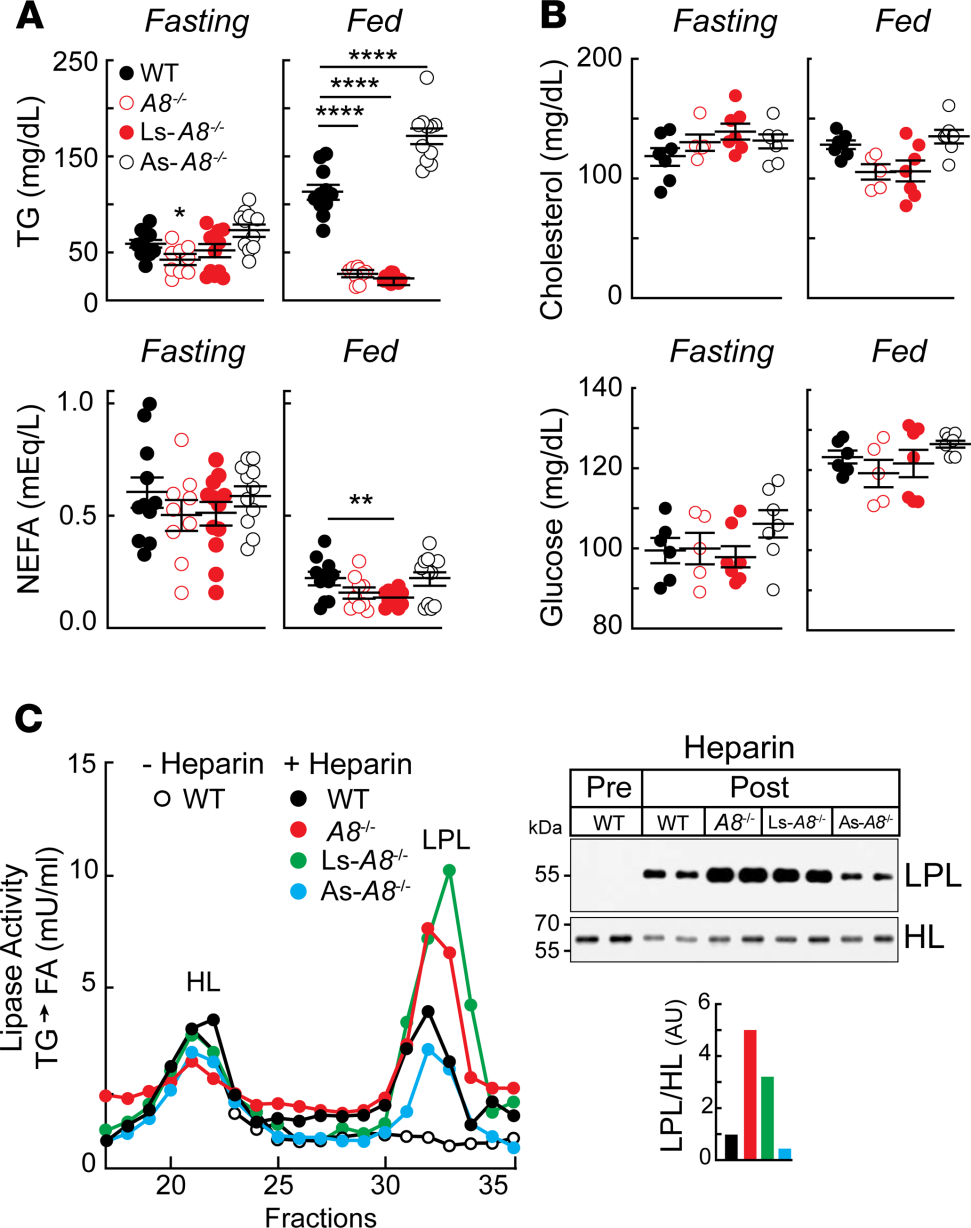
Plasma lipid and glucose levels, postheparin plasma lipase activities, and intravascular LPL levels in mice of the indicated genotypes. (**A**) Diets of age-matched male mice (*n* = 5–6/group, 10–15 weeks) were synchronized as described in the Methods. Plasma samples were obtained at the end of the last fasting period (Fasting) and then 4 hours after refeeding (Fed), and levels of TGs, nonesterified fatty acids (NEFAs) and (**B**) cholesterol were measured in duplicate (5 μL). Plasma glucose levels were measured from the first drop of blood from the tail vein. Values are shown as means (± SEM). The experiment was repeated and the data were similar. Groups were compared using 2-way ANOVA. *P < 0.05; **P < 0.01; ****P < 0.0001. The experiment was performed 3 times and the compiled results are shown. (**C**) The diets of the mice were handled as described in panel **A**. Four hours after refeeding, blood was collected from the WT mice. Mice were then injected with heparin intravenously (1 U/g), and blood was collected after 15 minutes. Pooled plasma was fractionated on a heparin column (1 mL) to separate hepatic lipase (HL) and LPL. TG lipase activity was measured in each fraction as described in Methods. Pooled plasma samples (10 μL) were diluted to 500 μL with PBS and incubated with heparin beads (20 μL) for 2 hours. The heparin bead–bound proteins were subjected to immunoblotting using anti-mouse LPL and HL polyclonal antibodies, as described in the Methods. Quantitative values are expressed as ratios to the level of HL and expressed relative to the WT value, which was set to 1.

As we have shown previously, inactivation of A8 has little impact on fasting levels of plasma TGs, cholesterol, and nonesterified fatty acids (NEFAs) (Figure 5, A and B) (although a modest, but marginally significant [*P* = 0.05] reduction in mean TG levels was seen in the A8-KO mice when compared with WT animals). These are expected findings because A8 is expressed at very low levels in fasting animals (11, 19, 20). Similarly, no changes in circulating fasting levels of cholesterol or glucose were seen in the tissue-specific A8-KO mice (Figure 5B).

In contrast to these findings, we observed significant differences in plasma TG levels between the groups after refeeding (Figure 5A). As expected (21), mean postprandial TG levels were significantly lower in *A8^–/–^* than in WT mice, whereas plasma levels of NEFAs (and cholesterol) were similar in these 2 groups of animals. Levels of plasma TGs were also reduced in the Ls-*A8^–/–^* mice. The reduction in mean levels of TGs in the plasma of these mice was similar to those of the *A8^–/–^* mice (23.1 ± 1.1 versus 27.6 ± 2.5 mg/dL). These findings are consistent with the notion that liver-derived A8, with A3, is responsible for inhibiting intravascular lipolysis in the refed state.

More surprising was the finding that plasma TG levels were increased in the fed As-*A8^–/–^* mice when compared with the WT animals (171.2 ± 8.3 versus 112.9 ± 7.3 mg/dL, *P* < 0.0001). To determine whether the increase in plasma TG levels in the As-*A8^–/–^* mice was caused by a decrease in intravascular lipolysis, we measured and compared heparin-releasable LPL activity among the strains. Four hours after eating, mice were injected with heparin (1 U/g). Blood was collected 15 minutes after heparin administration, and the plasma was subjected to heparin-affinity fast protein liquid chromatography fractionation to separate hepatic lipase (HL) and LPL (32). Figure 5C shows the lipolytic activities of the plasma fractions. Hepatic lipase activities were similar among the strains and did not change after heparin injection, as expected because HL circulates in plasma of mice (unlike humans) (33). Moreover, we showed previously that HL activity is not affected by A8 levels (21). In contrast to HL, LPL activities differed widely among the strains. LPL activity was increased to a similar extent in Ls-*A8^–/–^* and *A8^–/–^* mice when compared with As-*A8^–/–^* and WT animals. The increase in postheparin plasma LPL activity in *A8^–/–^* mice was comparable to that seen in *A3^–/–^* mice, which is consistent with the hypothesis that the 2 proteins act together to inhibit LPL (Supplemental Figure 2). In contrast to the increase in postheparin plasma LPL activity seen in Ls-*A8^–/–^* mice, As-*A8^–/–^* mice had an LPL activity that was similar to, or slightly lower than, that seen in WT animals (Figure 5C).

To determine whether the differences in LPL activity in our genetically modified mice were due to variations in the amount of enzyme on the endothelial surfaces, we compared levels of heparin-releasable LPL in the 4 strains. We incubated pooled postheparin plasma with heparin-Sepharose beads, eluted the bound proteins, and then subjected the eluted proteins to immunoblotting (Figure 5C, right). In general, the relative abundance of LPL in each strain corresponded with the relative LPL activity (Figure 5C, left). The relative abundance of LPL was similar in Ls-*A8^–/–^* and *A8^–/–^* mice and higher than that observed in the WT or As-*A8^–/–^* mice. The slightly reduced amount and activity of LPL in the As-*A8^–/–^* mice compared with the WT mice may contribute to the increase in circulating TG levels seen in these animals after refeeding (Figure 5A).

To rule out the possibility that the increase in plasma TG levels in the As-*A8^–/–^* mice was due to an increase in VLDL-TG entry into plasma, we monitored the increase in plasma TG levels after inhibiting intravascular LPL using Triton WR 1339 (34). As expected, the mean plasma levels of TGs were lower in the Ls-*A8^–/–^* mice at the start of the experiment. The rate of rise of TG levels was slower in the Ls-*A8^–/–^* mice than in the WT animals (5.57 mg/dL/min vs. 8.21 mg/dL/min, *P* < 0.01). In contrast to these results, no significant differences were found in the rate of TG increase between the As-*A8^–/–^* (9.60 mg/dL/min) and WT mice (Supplemental Figure 3).

### A model of the effect of hepatic A8 expression on TG trafficking and substrate usage in adipose tissue.

**Figure 6:**
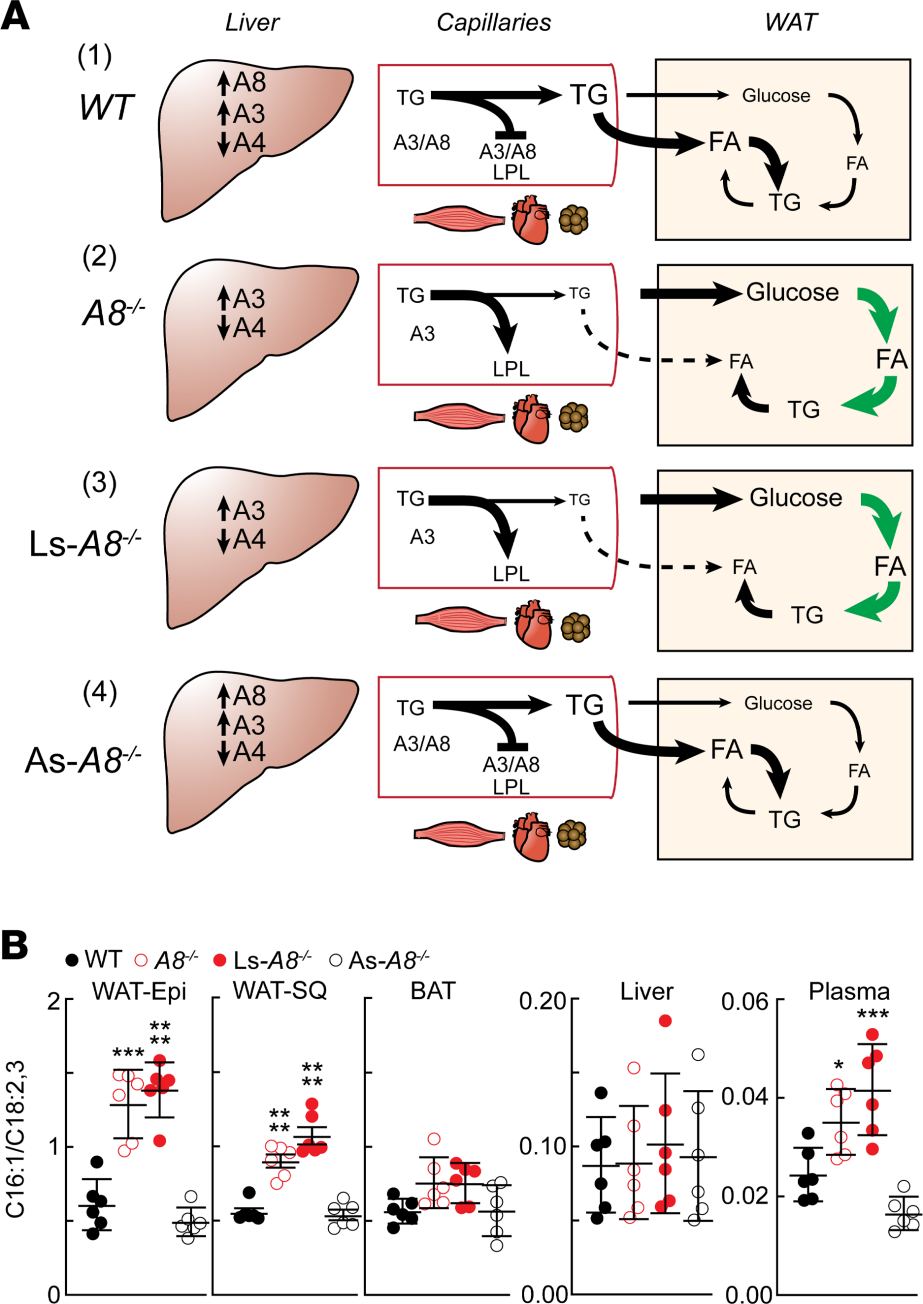
A schematic showing the role of hepatic A8 and A3 in TG trafficking in the fed state. (**A**) Expression of both A8 and A3 mRNA increase and A4 mRNA levels decrease with feeding (11). (1) A3 and A8 form a complex that inactivates intravascular LPL, which is most active in oxidative tissues. As a result, circulating TG is available for hydrolysis and uptake by adipose tissue. (2) Absence of A8 results in more TGs being available for uptake by oxidative tissues and less being available for uptake by adipose tissue. Glucose uptake, and its conversion to TG, are enhanced in adipose tissue of *A8^−/−^* mice. (3) TG trafficking in the Ls-*A8^−/−^* mice resembles that of the *A8^−/−^* mice (4) whereas TG trafficking in the As-*A8^−/−^* mice resembles that seen in the WT animals. (**B**) Levels of C16:1 (endogenously synthesized) and C18:2 and C18:3 (diet derived) in plasma and tissue lysates (adipose tissue and liver) were measured as described in the Methods, and the mean ratio (± SEM) of C16:1 to C18:2 plus C18:3 in each tissue is shown. Groups were compared using 1-way ANOVA with Dunnett’s multiple-comparisons test. *P < 0.05; ***P < 0.001; ****P < 0.0001.

To develop a framework for experimental testing, we revised our working model of how hepatic A8, with A3, coordinate TG trafficking and usage in the fed state (Figure 6A). In this model, feeding increases expression of A8 in liver. Liver-derived A8 forms a functional complex with A3 that inhibits LPL in oxidative tissues, thus reducing TG uptake in those tissues. A greater proportion of circulating TGs is now available for delivery to adipose tissue (Figure 6A). In the absence of A8, feeding fails to suppress LPL in oxidative tissues. Uptake of TGs into oxidative tissues remains high, and less TG is available for uptake by WAT (Figure 6A). Direct measurements of VLDL-TG delivery to tissues support this model (21). To compensate for the decreased delivery of TGs to WAT, glucose uptake into WAT is greatly increased. The additional glucose provides the substrate for fatty acid and TG synthesis to maintain energy stores (21).

Based on our findings here, selective inactivation of A8 in liver would be predicted to disrupt delivery of TGs to adipose tissue because of failure to inhibit intravascular lipolysis (Figure 6A). The reduction in TG delivery to WAT would result in an increase in both glucose uptake and endogenous synthesis of fatty acids in adipose tissue. Finally, in the As-*A8^–/–^* mice, TG trafficking would be expected to resemble that seen in the WT animals because both hepatic A3 and A8 are expressed in these animals (Figure 6A). The endocrine actions of A3/A8 on intravascular lipolysis ensure that sufficient TGs are delivered to WAT to maintain tissue energy stores.

To test these predictions, we compared the ratio of C16:1, a fatty acid that is absent from the diet and must be synthesized endogenously (35), to C18:2 and C18:3, which are entirely diet derived, in several tissues. As expected, the ratio of endogenous/exogenous fatty acids increased in the WAT-Epi and WAT-SQ, and to a lesser extent in BAT of the *A8^–/–^* and Ls-*A8^–/–^* mice (Figure 6B). The increased ratio of endogenous/exogenous fatty acids was also noted in the circulating free fatty acid pool of fasting animals, though absolute and relative abundance of C16:1 was much lower in plasma than in adipose tissue (note differences in *y* axis scale). In contrast, the ratio of endogenous/exogenous fatty acids in the As-*A8^–/–^* mice was similar to that of WT animals in all tissues sampled. Thus, delivery of dietary fats to WAT is independent of adipose tissue–derived A8 in the fed state. As expected, the ratio of endogenous/exogenous fatty acids in fasting plasma resembles that seen in the fed *A8^–/–^* and Ls-*A8^–/–^* mice (Supplemental Figure 5).

We used real-time PCR (oligonucleotide sequences provided in Supplemental Table 3) to measure levels of mRNAs encoding proteins involved in fatty acid synthesis and oxidation, glycolysis, beiging/browning in WAT-SQ, WAT-Epi, BAT, and liver (Supplemental Figure 4). The levels of mRNAs encoding enzymes involved in fatty acid synthesis were higher in the WAT of both the *A8^–/–^* and Ls-*A8^–/–^* mice. As shown previously, levels of sterol regulatory element-binding protein 1c mRNA were increased in the WAT of *A8^–/–^* mice but not in our tissue-specific KO strains despite an increase in fatty acid synthesis gene expression in the Ls-*A8^–/–^* mice. mRNA levels of another transcription factor that stimulates fatty acid synthesis, carbohydrate response element binding protein–β (36), were significantly elevated in WAT of both the *A8^–/–^* and Ls-*A8^–/–^* mice and increased, though not significantly, in the As-*A8^–/–^* mice (Supplemental Figure 4). We noted no differences in the mRNA levels of genes encoding proteins involved in fatty acid oxidation in WAT. Furthermore, uncoupling protein 1 mRNA levels were not significantly altered in the 4 mouse strains in any of the tissues tested. None of the transcripts implicated in beiging/browning, except Cell death-inducing DNA fragmentation factor-α-like effector A (CIDEA) and epithelial V-like antigen-1 (EVA1), differed among the strains (37). Levels of CIDEA and EVA1 transcripts were significantly higher in the *A8^–/–^* and Ls-*A8^–/–^* mice. Only minor changes were seen in the level of various hepatic mRNAs among the 4 strains of mice (Supplemental Figure 4D). These patterns of RNA expression are consistent with the hypothesis that fatty acid synthesis is upregulated in the WAT of *A8^–/–^* and Ls-*A8^–/–^*. Levels of the corresponding mRNAs were not increased in the livers of these mice. Therefore A3 and A8 secreted from the liver promote the efficient partitioning of fatty acids to WAT and thereby limit glucose uptake and de novo lipogenesis by this organ.

**Figure 7:**
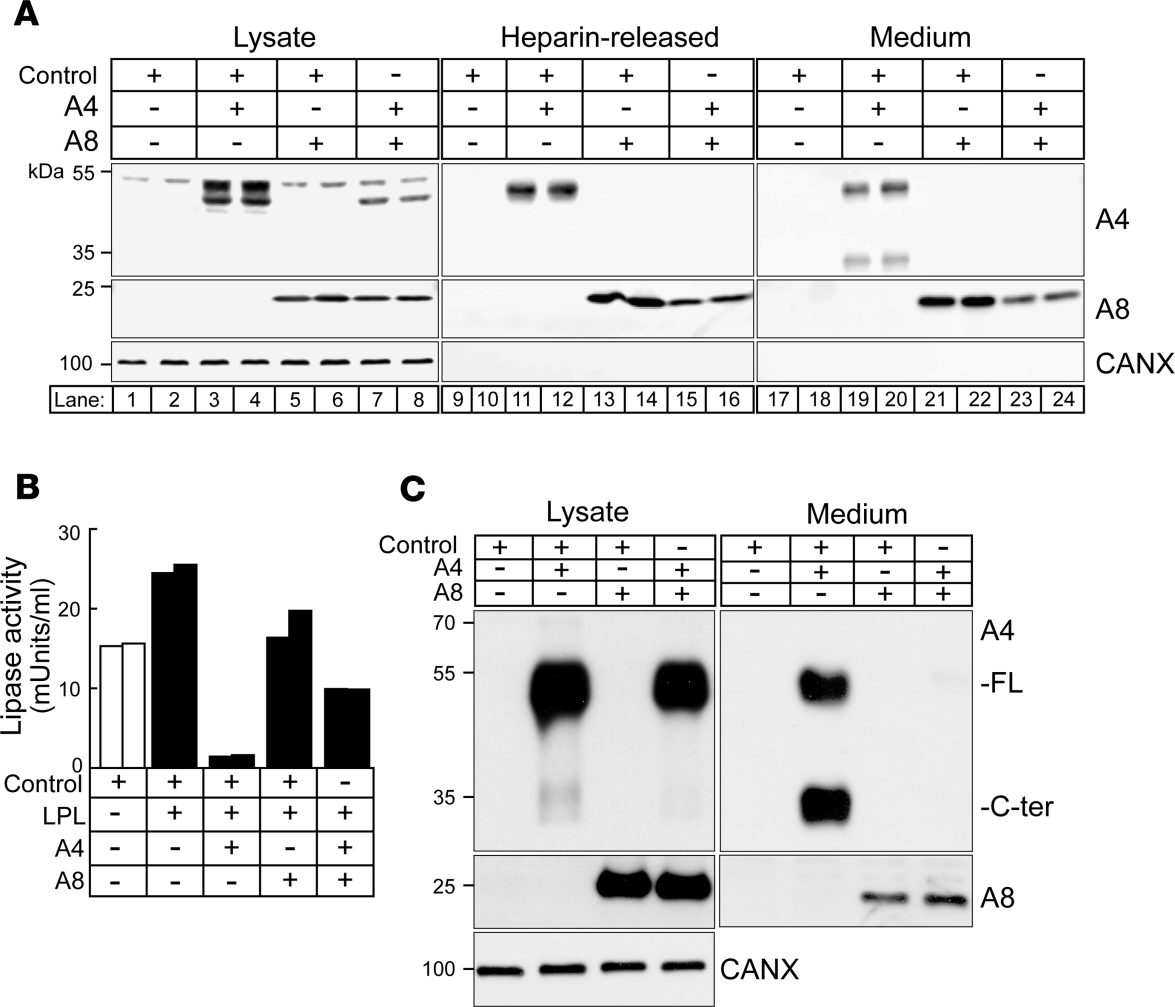
A8 prevents A4 secretion and attenuates A4 inhibition of LPL in CHO-K1 cells and in 3T3-L1 adipocytes. (**A**) Equimolar amounts of control (empty plasmid) and recombinant A4 and A8 were expressed in CHO-K1 cells alone or together. Whole-cell lysates, the heparin-released fraction, and conditioned medium were subjected to SDS-PAGE and immunoblotting using anti-A4 (top), anti-A8 (middle), and anti-calnexin antibodies as described in the Methods. (**B**) CHO-K1 cells were cotransfected with control, A4, A8, and LPL plasmids as indicated. The LPL activity was assayed in the medium 48 hours after transfection, as described in the Methods. (**C**) Differentiated 3T3-L1 adipocytes were infected with control and single or combination of A8 and A4 adenoviruses as described in the Methods. After 24 hours, cells and medium were collected, and immunoblot analysis was performed using anti-A4 (top), anti-A8 (middle), and anti-calnexin antibodies on cell lysates and cultured medium. The experiments were performed 2 additional times and the results were similar.

These observations raise the question as to the action of the adipose-specific A8 protein. One possibility is that A8 expression in adipocytes has local effects that include altering the synthesis or secretion of A4, the major regulator of LPL activity in WAT (38). Whereas mRNA levels of A4 and A8 are regulated reciprocally by fasting and feeding, Kroupa et al. showed that the turnover of A4 protein is very slow in WAT from young rats treated with actinomycin D (39). After 6 hours of treatment, levels of A4 mRNA were reduced by 93%, whereas levels of ANGPTL4 protein did not change. Thus, degradation of A4 appears to require the synthesis of new protein, possibly A8. We tested whether A8 promotes the degradation of A4 during the transition from fasting to refeeding, which would accelerate the activation of adipose tissue LPL in response to food intake. We tested this hypothesis by examining A4 synthesis and secretion in transiently transfected cultured cells expressing A4 and A8 (Figure 7A, top).

### A8 expression reduces secretion of A4 from cultured cells.

Recombinant A4 was expressed in the presence and absence of A8 in CHO-K1 cells. A doublet corresponding to the sizes of full-length, mature A4 (50 kDa) and the immature, unglycosylated precursor form of A4 (46 kDa) was seen in cells expressing A4 alone (Figure 7A, lanes 3 and 4) (22, 40). Expression of A4 with A8 caused a reduction in the levels of A4, but not A8, in cell lysates (lanes 7 and 8). Cells were treated with heparin to analyze cell surface proteins. Bands corresponding to full-length A4 and A8 were seen in cells expressing either A4 or A8 alone after heparin treatment (Figure 7A, lanes 11 and 12 or 13 and 14, respectively). However, in cells coexpressing A4 and A8, no heparin-releasable A4 protein was detected (lanes 15 and 16). This result is consistent with a scenario in which A8 expression promotes the intracellular degradation of A4.

To rule out the possibility that the absence of A4 on the surfaces of cells cotransfected with A8 was due to displacement of A4 by A8 on the cell surface, we examined the medium for the presence of A4. Bands of the expected size for the full-length and C-terminal domain of A4 were present in the medium of cells expressing A4 alone (lanes 19 and 20). Thus, a portion of the A4 undergoes catalytic cleavage during or after its secretion, as described previously (41). In contrast, when A8 and A4 were coexpressed, no full-length or cleaved A4 was detected in the medium (lanes 23 and 24). Thus, A8 expression dramatically reduces secretion of A4 from cells. The levels of A8 were 50% lower in the medium of cells coexpressing A8 and A4 than in cells expressing A8 alone (compare lanes 23 and 24 with lanes 21 and 22). Thus, A4 coexpression seemed to have a more tempered effect on secretion of A8.

Next, we examined the effect of A4 and A8 on LPL activity in the medium of CHO-K1 cells (Figure 7B). These cells had significant endogenous LPL activity, as indicated by the high lipase activity in cells expressing the control plasmid (column 1). Transfection with recombinant LPL plasmid further increased LPL activity (column 2). However, coexpression of LPL with A4, but not with A8, resulted in a dramatic reduction in LPL activity to almost undetectable levels, as had been observed previously (columns 3 and 4, respectively) (42). Coexpression of A4 and A8 in cultured cells attenuated the inhibitory effect of A4 expression on lipase activity (column 5). Taken together, these results suggest that A8 interferes with secretion of A4 in cells, which serves to attenuate the LPL inhibitory action of A4. However, we cannot exclude the possibility that A8, synthesized in the liver or adipose tissue, also acts in the subendothelial space.

To confirm that the observations made in cultured CHO cells resembled those seen in adipocytes, we used adenoviruses to express A4 and A8 in differentiated 3T3-L1 adipocytes (Figure 7C). The results were consistent with our findings in CHO-K1 cells: expression of recombinant A4 with A8 resulted in a significant reduction in A4 secretion. Conversely, A4 expression had little to no effect on A8 expression or secretion.

### A8 physically interacts with A4 when coexpressed in cultured cells.

**Figure 8:**
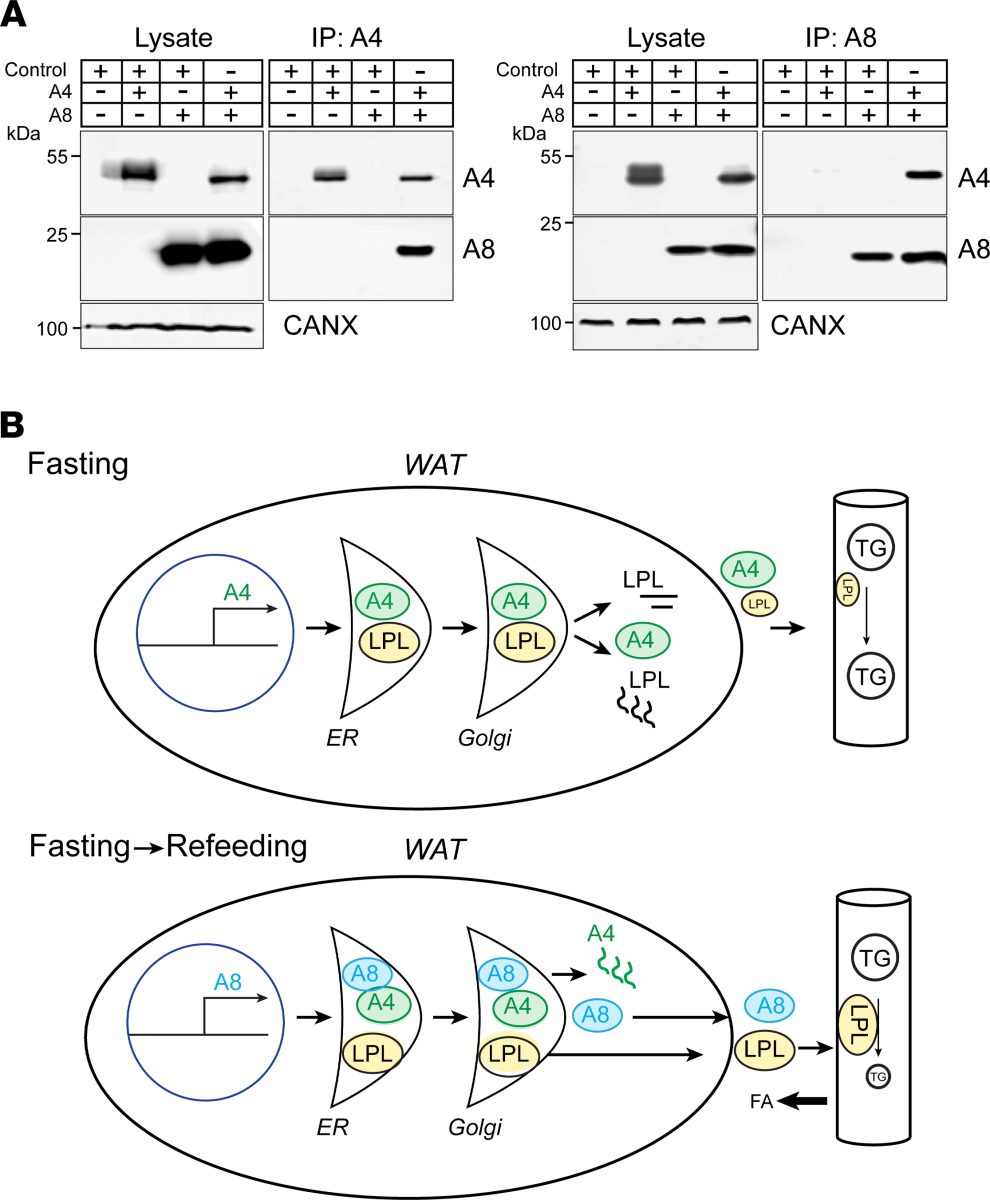
A8 inhibits A4-mediated LPL degradation by interacting with A4. (**A**) A8 physically interacts with A4 in QBI-293 cells. Recombinant A4-Myc and A8-Flag were coexpressed in QBI-293 cells alone (with empty plasmid) or together. A4 was immunoprecipitated using Myc-linked magnetic beads (left panel), and A8 was immunoprecipitated using Flag magnetic beads (right panel) as described in the Methods. As A4 has nonspecific binding to magnetic beads, Flag beads were eluted using 3× Flag peptide. Immunoblot analysis using anti-A4 (top), anti-A8 (middle), and anti-calnexin antibodies was performed using 30 μg of lysate (input) as described in Methods. (**B**) Schematic model of A4, A8, and LPL expression in WAT from fasted (top) and refed (bottom) mice. After a 15-hour fast, no A8 is present in the adipose tissue of WT mice whereas levels of expression of A4 are high. A4 expression promotes degradation of LPL. As a consequence, circulating lipoproteins bypass WAT and deliver TGs to oxidative tissues (top). In the fasting to feeding transition, A8 expression rises as levels of A4 slowly fall. A8 interacts with A4, thus sparing LPL from A4-stimulated intracellular degradation. LPL is now available to hydrolyze circulating lipoprotein-TGs and thus replete TG stores in adipose tissue when food is available.

Genetic ablation of A8 in mouse livers resulted in increased hepatic expression of A3 (Figure 3) while expression of A8 in differentiated 3T3-L1 adipocytes decreased expression of A4 (Figure 7C). These findings are consistent with a model in which interaction with A8 in the secretory pathway promotes the intracellular degradation of both A3 and A4. To test for interaction between A4 and A8, we expressed full-length recombinant epitope-tagged constructs of A4 (A4-Myc) and A8 (A8-Flag) in QBI-293 cells and then immunoprecipitated A4 from the cell lysates (Figure 8A). In cells coexpressing A4-Myc and A8, an anti-Myc antibody precipitated both proteins. As expected, the anti-Myc antibody failed to immunoprecipitate A8 from cells expressing A8 alone. Moreover, when the reverse experiment was performed and A8 was immunoprecipitated from cells, A4 was also pulled down with A8 (Figure 8A). Thus, A4 and A8 physically interact in cells expressing both proteins. We performed the same coimmunoprecipitation experiment in CHO-K1 cells, and the results were similar (Supplemental Figure 6).

## Discussion

The major finding of this study is that the atypical angiopoietin-like protein, A8, has distinct functions in liver and adipose tissue. In both tissues, A8 acts by altering the activity of 2 canonical ANGPTLs: A3 in liver and A4 in adipose tissue. Selective inactivation of liver A8 resulted in mice with no circulating A8 (Figure 3A), high intravascular LPL activity, low plasma levels of TGs, and increased endogenously synthesized fatty acids in WAT. These data indicate that essentially all circulating A8 originates in the liver and that the postprandial inhibition of LPL activity in oxidative tissues, the major site of action of LPL, is mediated by a complex between liver-derived A8 and A3. Inactivation of A8 in adipose tissue had little to no effect on postheparin plasma LPL activity and was associated with an increase rather than decrease in the level of circulating TG. Thus, A3 and A8 from liver, and A8 from adipose tissue, use different mechanisms to control uptake and utilization of energy substrates by tissues in response to food intake.

The effects of liver-specific inactivation of A8 on plasma TG levels (Figure 5A) and on adipose fatty acid composition (Figure 6B) were remarkably similar to those seen in the *A8^–/–^* mice (7). Other than promoting intravascular LPL inhibition (Figure 5), A8 expression in liver is associated with a reduction in A3 secretion. WT and As-*A8^–/–^* mice had lower levels of full-length A3 (but not the cleaved fragments of A3) in both plasma and liver lysates than either the Ls-*A8^–/–^* or total *A8^–/–^* mice (Figure 3, A and B). By reducing A3 secretion, A8 may indirectly alter the activity of another lipase, endothelial lipase (EL) (43), since A3 inhibits EL (44) and this activity does not require A8.

Full-length A3 levels were increased in the plasma and liver of the *A8^–/–^* and the Ls-*A8^–/–^* mice, without an increase of similar magnitude in A3 mRNA, suggesting that A8 expression is associated with reduced secretion of A3 from the liver. Very low levels of C-terminal or N-terminal fragments of A3 were visible in liver lysates, yet levels of the 2 fragments in plasma were similar among the 4 strains (Figure 3). These results are consistent with cleavage of A3 occurring after its secretion from liver. Additional experiments will be required to determine why levels of the A3 fragments do not differ between the strains of mice.

Unlike the effect of A3 on EL, which does not require A8, the inhibitory effect of A3 on LPL activity is markedly enhanced in the presence of A8 (25). The results of in vitro studies suggest that A8, rather than A3, mediates the physical interaction between the A3/A8 complex and LPL (25). Disruption of the LPL binding site of A3 does not attenuate the inhibitory effect of the A3/A8 complex on LPL. Although detailed biophysical studies of the interaction between the A3/A8 complex and LPL have not been performed, we would predict that A3/A8 binding to LPL results in degradation of the protein. Consistent with this prediction, the amount of LPL in postheparin plasma of *A3^–/–^* and *A8^–/–^* mice was increased to a similar extent and was proportional to the increase in LPL activity (Supplemental Figure 2). Moreover, A8 from liver, but not from adipose tissue, conferred this effect on LPL (Figure 5C).

Although the Ls-*A8^–/–^* mice recapitulated the intravascular phenotype of the *A8^–/–^* mice, inactivation of A8 in the liver alone did not cause a reduction in body weight or fat pad mass (Figure 1B and Figure 2B). This finding implies a role for A8 expressed in adipose tissue in the maintenance of TG stores. How is this accomplished? In contrast to hepatocytes, adipocytes do not appear to contribute to the A8 plasma pool (Figure 3A). The As-*A8^–/–^* mice had similar, albeit slightly lower, postheparin LPL levels and activity (Figure 5C), which may contribute to the higher plasma TG levels (Figure 5A) seen in these animals when compared with WT mice. Our data suggest that A8 in adipose tissue antagonizes the inhibitory effect of A4 on LPL activity. A8 synthesis (and A4 inhibition) in adipose tissue in the early fasting-refeeding transition would increase LPL activity in adipose tissue and enhance the rate at which TG stores are replenished, thus better preparing the animal for the next fast (Figure 8B).

The accumulation of C16:1 and depletion of C18:2 in WAT of *A8^–/–^* mice is consistent with our previous finding that inactivation of A8 prevents the postprandial increase in the uptake of circulating TGs that replenishes WAT in WT animals. A similar elevation in the ratio of C16:1 to C18:2/18:3 was seen in WAT from the Ls-*A8^–/–^* mice, but not in As-*A8^–/–^* mice. These data indicate that the uptake of circulating TG-fatty acids by WAT is controlled by endocrine actions of A8 from the liver, rather than by the adipocytes themselves. Despite similar increases in C16:1 to C18:2/18:3 ratios, the mass of the epididymal fat pads was markedly reduced in *A8^–/–^* mice, but not in the Ls-*A8^–/–^* mice. Thus, the increases in glucose uptake and de novo fatty acid synthesis that drive the changes in fatty acid composition in the WAT of these animals is sufficient to preserve WAT in the Ls-*A8^–/–^* mice but not in the *A8^–/–^* mice. Ls-*A8^–/–^* mice resemble the adipose tissue–specific LPL KO in that they have increased de novo lipogenesis in adipose tissue, as reflected by the changes in fatty acid composition (45). Taken together, these data indicate that expression of A8 either in liver or in adipose tissue is sufficient to preserve WAT mass in mice.

Figure 8B depicts our current model of LPL regulation by A4 and A8 in WAT. In fasting animals, the levels of A4 in WAT are high, whereas those of A8 are very low (Figure 8B, top). A4 is a potent inhibitor of LPL: prior studies in cultured cells showed that coexpression of recombinant A4 and LPL promotes the intracellular degradation of LPL, although the mechanism by which this is accomplished remains unclear (42). Consequently, LPL activity is low in WAT during fasting, causing circulating TG to bypass WAT in favor of oxidative tissues where LPL activity is high.

Conversely, A8 expression is induced upon feeding (Figure 8B, bottom) physically interacts with A4 in adipocytes, leading to its degradation. This finding is consistent with the data of Kovrov et al. (14), who reported that purified recombinant A8 can form complexes with the N-terminal domain of A4 if the proteins are refolded together from their denatured state. Under those conditions, A8 decreased the inhibitory effect of A4 on LPL activity by about 50%. If A8 has the same effect on A4 secretion in vivo in adipose tissue as it does in cultured adipocytes (Figure 7C), then A8 expression in WAT would reduce the LPL inhibitory effect of A4 and increase LPL activity in adipose tissue. In short, A8 would act locally in WAT to enhance the uptake of TGs into adipocytes upon feeding, leading to a more rapid repletion of energy stores. Based on this model, we predict that the trafficking of LPL is similar in fasting WT and As-*A8^–/–^* mice (Figure 8B, top) because A4 promotes intracellular degradation of LPL, resulting in less LPL in the intravascular space. In both strains, circulating TGs would largely evade hydrolysis in WAT and be preferentially delivered to oxidative tissues. We provide evidence that A8 within adipocytes acts inside the cell to attenuate the inhibitory effect of A4 on LPL activity. However, we cannot exclude a potential action of A8 in the subendothelial space too. Because we show that A8 physically interacts with A4, it is also plausible that circulating A8 could block the extracellular endothelial cell–associated effects of A4. Taken together, these findings are compatible with a model in which A8 has a dual role that is dependent on its site of expression and on its stoichiometric relationships with A3 and A4. In liver, A8 activates A3 to form an A3/A8 heterodimer. The A3/A8 heterodimer acts as an endocrine factor that mediates postprandial rerouting of TGs from oxidative tissues to adipose tissue. In adipose tissue, A8 interacts instead with A4 and functions in an autocrine or paracrine fashion to facilitate energy storage in adipocytes.

The most perplexing phenotypes seen in the tissue-specific *A8^–/–^* mice are the increases in plasma TGs and in body temperature in the As-*A8^–/–^* mice when compared with WT animals (Figure 1C and Figure 5A). The increase in plasma TGs in As-*A8^–/–^* mice cannot be explained by previous models from our laboratory or others (21), which assumed that a single pool of circulating A8 acts with A3 to inhibit LPL in the circulation. If adipose tissue does not contribute to the intravascular pool of A8 (Figure 3), why does inactivating A8 in that tissue cause an increase in plasma TGs (Figure 5A)? We have ruled out the possibility that inactivating A8 in mice increases plasma TGs by increasing VLDL-TG secretion (Supplemental Figure 3; see also complete unedited blots in the supplemental material). It may be that the absence of A8 in adipocytes results in greater A4 activity and decreased secretion of LPL, and hence increase in TGs in the fed state. Additional experiments will be required to test this hypothesis.

Basic mechanistic questions remain even for A4, the best studied of the 3 ANGPTL proteins. The Neher laboratory proposed that A4 binds the lid domain of LPL and occludes access to the catalytic site (46). In this model, inhibition of LPL activity is reversible and does not involve denaturation or dissociation of the enzyme. The Olivecrona laboratory reported that A4 promotes irreversible dissociation of dimeric LPL into catalytically inactive monomers (38). Inactivation of LPL occurred at substoichiometric ratios of A4, which was not consumed (38). Using deuterium exchange, Mysling et al. (47) also found that A4 inactivates LPL at substoichiometric ratios and that binding of A4 to LPL results in irreversible unfolding of the protein. Studies by the same group showed that A3 had a similar but much weaker effect (47). Although further biochemical studies will be required to elucidate the specific mechanism by which A3/A8 inhibits LPL, the increase in both the activity and the amount of LPL in postheparin plasma from A8-KO mice indicates that A3/A8 binding to LPL results in depletion of the enzyme from the capillary endothelial surfaces. These data favor a model in which A3/A8 binding to LPL results in irreversible inactivation, possibly via unfolding of LPL (47) and degradation of the enzyme.

*A8^–/–^* mice are hypermetabolic for reasons that remain to be elucidated (28). These effects were more pronounced in the *A3^–/–^ A8^–/–^* mice but were absent in the *A3^–/–^* mice (28). We noted differences between the C57BL/6J background of *A8^–/–^* mice we studied previously and the *A8^–/–^* mice used in the experiments described in this paper, which were in a different substrain of C57BL/6 (C57BL/6N). On a C57BL/6J background, the mice had an increase in VO_2_ consumption, which we did not see in the C57BL/6N mice (Supplemental Figure 1C). The reason for these differences may be the well-documented differences in substrate usage between these 2 strains of mice (29). Alternatively, the differences may be due to environmental factors that have yet to be identified.

An increased body temperature in response to eating was apparent in both *A8^–/–^* and Ls-*A8^–/–^* mice. In both strains, no difference in body temperature was apparent in the fasting state, only after refeeding (Figure 1C) and only if the mice were maintained at a temperature lower than thermoneutrality (Supplemental Figure 1C). Previously, we showed that we could normalize the body temperature of *A3^–/–^ A8^–/–^* mice by inhibiting the β_3_-adrenergic receptor, which is only expressed in adipocytes (48). These results suggested that the effect of A8 inactivation on body temperature was due to A8 expression in adipose tissue, not the brain function. Here we did not see any postprandial increase in body temperature in Ls-*A8^–/–^* animals, which further implicates A8 expression in the adipose tissue being responsible for changes in body temperature. Our data do not implicate A8 expression in BAT as a major contributor to the phenotypes observed in our tissue-specific KO mice. These observations are consistent with our previous findings (7, 28). We showed that A8 physically interacts with A4 (Figure 8A) and reduces its secretion in cultured adipocytes (Figure 7C). Thus, absence of A8 in adipose tissue would be expected to cause an increase in the secretion of A4. A4 undergoes catalytic cleavage, releasing the N- and C-terminal fibrinogen-like domains. The C-terminal fibrinogen-like domain of A4 has been shown to activate the β_3_-adrenergic receptor signaling pathway (49), which we showed previously plays a critical role in the hyperthermia associated with A8 inactivation (28). Additional studies will be required to determine how A8 suppresses postprandial increases in body temperature.

## Methods

### Mice.

The genetically modified mice used in these studies were in a C57BL/6NTac genetic background. The Alb-*Cre* transgenic mice were obtained from The Jackson Laboratory and were on a C57BL/6N background (strain 018961). The Adipo-*Cre* mice were provided by Philipp Scherer (UTSW) and backcrossed 7 generations with C57BL/6N from Charles River before being bred to the genetically modified A8 mice. The *fl/fl* mice were obtained from Regeneron Pharmaceuticals. To generate hepatocyte (referred to as Ls-*A8^–/–^* mice) and adipocyte (As-*A8^–/–^*) mice, the *fl/fl* mice were crossed with Alb-*Cre* and Adipo-*Cre* mice, respectively. The *A8^−/−^* mice were generated as described (11, 21, 50) and backcrossed with C57BL/6N mice for 7 generations. Mice were maintained at 21°C–23°C on a 12-hour light/12-hour dark cycle (lights on: 6:00 am to 6:00 pm) and fed a rodent chow diet (Teklad Global, 16% protein). For all experiments, unless otherwise stated, the feed/fast cycles of the mice were synchronized for 3 days by removing food during the night for 15 hours (6:00 pm to 9:00 am) and providing food at 9:00 am. Samples were collected after the 15-hour fast (fasting) or 4 hours after the mice were provided food (fed).

Mice were placed in metabolic cages (LabMaster System; TSE Systems) for 3 days before measuring food intake and physical activity, which were monitored for 1 minute every 50 minutes for 4 days. Indirect calorimetry was performed to measure VO_2_ consumption and VCO_2_ output. Measurements of body composition, including fat mass, lean tissue mass, free water, and total body water, were performed in nonanesthetized mice using NMR (Bruker Minispec MQ10), as previously described (7).

Rectal temperatures were measured using a thermocouple thermometer probe (BAT-7001H; Physitemp Instruments) that was inserted at least 1.5 cm into the rectum.

### Biochemical analysis.

Blood was collected from tail veins in citrate-EDTA tubes, and plasma was isolated by centrifugation at 2000*g* for 10 minutes at 4°C. Plasma levels of TGs and cholesterol were measured using Infinity series kits (Thermo Fisher Scientific). NEFA levels were measured in duplicate or triplicate using the HR Series NEFA-HR Kit (Fujifilm Wako Diagnostics). Results were obtained using the Synergy Neo2 plate reader (BioTek Instruments). Plasma glucose levels were measured using Contour Blood Glucose Monitoring System (Bayer).

For fatty acid analysis of tissues, lipids were extracted from the blood (51) of the fasting and refed animals, derivatized to form methyl esters, and separated by gas/liquid chromatography using a Hewlett Packard 6890 Series GC system. The identity of the fatty acid methyl ester was determined by comparing the retention times with fatty acid standards (GLC-744; NU-Chek Prep). The abundance of each fatty acid was determined from the peak intensity and the internal standard. Fatty acid profiles were generated using a modified GC-MS method, as previously described (52).

### Cell culture.

3T3-L1 murine fibroblasts (provided by the Department of Molecular Genetics, UTSW) were propagated and differentiated to adipocytes as described previously (53).

CHO-K1 cells (epithelial cells, provided by the Department of Molecular Genetics, UTSW) were cultured in DMEM/F12 with FBS (10%) and penicillin-streptomycin (1%). Myc-tagged human A4, Flag-tagged human A8, V5/His6-tagged human LPL, and empty vector pcDNA3.1 were transfected into CHO-K1 cells according to the manufacturer’s instructions (FuGENE 6 Transfection Regent, Promega). After 48 hours, the cell culture medium was collected and LPL activity was assayed. Cells were first washed with cold PBS once and then incubated on ice with 0.5 mL of PBS + heparin (50 U/mL) for 20 minutes on ice to release surface-bound LPL. The heparin-released fraction was removed and assayed for lipase activity. Cells were suspended in 300 μL of 20 mM Tris at pH 7.5, 150 mM NaCl, and 5 U/mL heparin and then homogenized by sonication 3 times for 5 seconds each. After removal of insoluble material, the supernatant was loaded onto SDS-PAGE and subjected to Western blotting.

QBI-293 cells (human kidney cells, provided by the Department of Molecular Genetics, UTSW) were cultured in DMEM with FBS (5%) and penicillin-streptomycin (1%). The protocol for transfection of QBI-293 cells was identical to that described for CHO-K1 cells.

### Immunoprecipitation.

QBI-293 cells were cultured in 100 mm dishes and transfected with Myc-tagged human A4, Flag-tagged human A8, and empty vector pcDNA3.1. Cells were collected 48 hours posttransfection and suspended in 1 mL of radioimmunoprecipitation assay (RIPA) buffer (25 mM Tris-HCl at pH 7.6, 150 mM NaCl, 1% NP-40, 1% sodium deoxycholate, 0.1% SDS) plus protease inhibitor. The cells were homogenized by sonication 3 times for 5 seconds each. After removal of insoluble material, the protein concentration was quantified by Bicinchoninic Acid Assay (Thermo Fisher Scientific). Lysates containing 800 μg total protein were incubated with 20 μL of prewashed anti-FLAG M2 magnetic beads or anti-Myc magnetic beads for 3 hours at 4°C. For A4 immunoprecipitation, anti-Myc beads were washed 3 times with RIPA buffer and resolved in 50 μL of protein loading buffer. A total of 20 μL was loaded onto SDS-PAGE and subjected to immunoblotting. For A8 immunoprecipitation, Flag beads were washed 3 times with RIPA buffer and then incubated with 50 μL of 1 mg/mL 3× Flag peptides in TBS buffer (25 mM Tris-HCl at pH 7.5, 150 mM NaCl) for 20 minutes at room temperature. A total of 20 μL of the total elution was loaded onto SDS-PAGE and analyzed by immunoblotting.

### SDS-PAGE and immunoblot analysis.

Tissues were collected from mice after a 15-hour fast or after refeeding for 4 hours. The tissues were snap-frozen in liquid nitrogen and stored at –80°C. Lysates of WAT were prepared using the Minute Total Protein Extraction Kit for Adipose Tissues/Cultured Adipocytes according to the manufacturer’s protocol (Invent Biotechnologies). Protein concentrations were determined using the Bio-Rad Bradford protein assay according to the manufacturer’s protocol. Mouse plasma was diluted 10-fold in 0.9% NaCl and then incubated at 85°C for 3 minutes in Laemmli SDS reducing sample buffer (375 mM Tris-HCl, 9% *w/v* SDS, 50% glycerol, 9% β-mercaptoethanol, 0.03% bromophenol blue, pH 6.8). A total of 10 μL of diluted plasma was size-fractionated on a 15% SDS-PAGE at 125 V and transferred to nitrocellulose (GE Healthcare). Membranes were incubated in PBST buffer (1× PBS, 0.1% Tween-20, pH 7.4) with 5% fat-free milk (Carnation) for 60 minutes at room temperature before overnight incubation at 4°C with antibodies (Abs) diluted in PBST buffer plus 5% fat-free milk. The following polyclonal Abs were used for immunoblotting: rabbit anti–fibronectin Ab (Abcam, Ab2413, 1:5000), goat anti–mouse A3 Ab (AF136, R&D Systems, Bio-Techne, 1:1000), and rabbit anti–calnexin Ab (Thermo Fisher Scientific, ADI-SPA-860-F, 1:3000). Mouse anti–A8 monoclonal Ab (IgG-19H4, 1:500) was produced by injecting New Zealand black mice 7 times with full-length recombinant mouse A8. Positive clones were screened by ELISA, confirmed by immunoblotting, and subcloned by serial dilution. The final subclone was expanded and the cell culture supernatant purified on a Protein G (4 Fast Flow) gravity column.

After incubation with the Ab, membranes were washed 3 times for 10 minutes in PBST buffer before adding horseradish peroxidase–conjugated goat anti–mouse or anti–rabbit IgG (1:10,000) (Thermo Fisher Scientific, 45000692 and 45000682) in PBST plus 1.25% fat-free milk. Following a 60-minute incubation at room temperature, membranes were washed with PBST and visualized using SuperSignal-enhanced chemiluminescence (Pierce, Thermo Fisher Scientific) before being exposed to F-BX810 Blue X-Ray films (Phoenix Research Products). Band intensities were analyzed using LI-COR Image Studio Lite version 5.2.5. For detection of human A4 and A8 in cell lysates and media, rabbit polyclonal anti–human A8 (149D) was used. 149D Ab was produced by injecting rabbits 10 times with His-tagged full-length protein in Freund’s adjuvant.

For detection of LPL and HL in mouse plasma, pooled plasma samples (10 μL) were diluted in 500 μL of PBS. The diluted samples were incubated with heparin beads (20 μL) for 2 hours. The heparin bead–bound proteins were then subjected to SDS-PAGE and Western blotting. The following Abs were used for immunoblotting: rabbit polyclonal anti-LPL polyclonal Ab (AF7197, R&D Systems, Bio-Techne) and rabbit anti-mouse HL polyclonal antibody (LIPC, LSBio). Ponceau staining was used for detection of proteins in the media (MilliporeSigma).

### Expression of recombinant human A4 and A8 in differentiated murine adipocytes (3T3-L1 cells).

Recombinant adenoviruses (1 × 10^9^ viral particles) expressing human A4 and A8 (11, 54) were used to infect differentiated adipocytes. The medium was replaced with serum-free medium 2 hours postinfection. After 24 hours, the medium was collected and subjected to centrifugation for 5 minutes (3500*g*, room temperature). The supernatant was concentrated 40-fold using Centrifugal Filters (Amicon Ultra, 10 mL–10 K; MilliporeSigma). Cell lysates were prepared using the Minute Total Protein Extraction Kit for Adipose Tissues/Cultured Adipocytes according to the manufacturer’s protocol (Invent Biotechnologies).

### Real-time PCR assay of mRNA levels.

Total RNA was extracted from tissues (*n* = 4–6 per genotype) using QIAGEN RNeasy Plus Universal Mini Kit (catalog 73404). Complementary DNA was obtained from 2 μg of total RNA using TaqMan (Applied Biosystems, Thermo Fisher Scientific) with random hexamer primers. Oligonucleotides specific for each transcript (Supplemental Table 3) were used to amplify from total RNA by quantitative PCR in 2× SYBR Master Mix (Applied Biosystems, Thermo Fisher Scientific) according to the manufacturer’s instructions. mRNA levels were normalized to levels of mouse 36B4 (liver), cyclophillin (WAT-Epi and BAT), and HPRT (WAT-SQ) transcripts. For each transcript, the mean level of the WT animals was set to 1.

### Intravascular lipase activity.

Heparin-Sepharose chromatography was performed using an ÄKTA purifier system (GE Healthcare, now Cytiva). Briefly, a HiTrap Heparin HP 1 mL column was equilibrated with buffer A (20 mM Tris-HCl, 0.15 M NaCl, 1% *w/v* BSA, and 20% *v/v* glycerol). A total of 250 μL of plasma pooled from 5 mice was applied to the column. The column was washed with buffer A and eluted with a linear gradient of NaCl (from 0.15 to 2.0 M) in buffer A (38). LPL activity was measured using a commercial LPL assay kit (STA-610, Cell BioLabs, Inc.). Adipose tissue (100 mg) or other tissues (50 mg) were homogenized in 1 mL of cold buffer A (20 mM Tris pH 7.5, 150 mM NaCl) and then centrifuged for 10 minutes at 10,000*g*. After incubation of tissue lysate or diluted plasma (10 μL) with reagents from the assay kit according to the manufacturer’s instructions, the fluorescence signal (excitation in 480–485 nm and emission in 515–525 nm) was read and recorded with a fluorescence microplate reader (Synergy Neo2, BioTek Instruments). LPL activity was calculated following instructions provided in the kit.

### Histology.

Following an overnight fixation in paraformaldehyde (4%), tissues were washed with PBS and processed for paraffin embedding. Tissues were sectioned to a thickness of 5 μm and stained with H&E as described previously (28). Stained sections were examined using light microscopy (original magnification, ×20). For each group, tissues from 5–6 mice were collected and stained. Representative images are shown.

### Rectal temperature, indirect calorimetry, and body composition measurements.

We recorded rectal temperatures in fasting and refed mice as described above. For measurements of energy homeostasis, mice were acclimated to the new environment for 3 days before data collection. Measurements of body composition, including fat mass, lean tissue mass, free water, and total body water, were performed in nonanesthetized mice by NMR (Bruker Minispec MQ10).

### VLDL-TG secretion measurements.

Mice were injected with a bolus Triton WR-1339 (500 mg/kg) (tyloxapol; MilliporeSigma) via the tail vein. Blood was collected from the tail vein at several time points, and plasma levels of TGs were determined using a colorimetric assay (Infinity Series Kit, Thermo Fisher Scientific).

### Statistics.

All results are expressed as mean ± SEM. Differences among groups were analyzed by unpaired 2-tailed Student’s *t* test, 1-way ANOVA followed by Dunnett’s multiple-comparisons test, or 2-way ANOVA for multiple comparisons. Analyses were performed using GraphPad Prism version 8.1.1 (GraphPad Software). Power calculations were performed using available online sources (http://www.biomath.info/power/ttest.htm). The sample sizes used in indirect calorimetry studies (*n* = 7 mice/group) were estimated to provide 90% power to detect differences in VO_2_ and VCO_2_ of 1.5 SD (equivalent to 300 mL/kg/h). For rectal temperature measurements, a sample size of 7 was estimated to 90% power to detect a difference in the means of 0.6°C. For all experiments, a *P* value of less than 0.05 was considered statistically significant.

### Study approval.

All protocols were approved by the Institutional Animal Care and Use Committee of the University of Texas Southwestern Medical Center.

## Author contributions

FO, HC, JCC, and HHH designed the experiments, and FO and HC performed the experiments. SB and VG contributed new reagents/analytic tools; FO, HC, JCC, and HHH analyzed data; and FO, HC, SB, GV and JCC, and HHH wrote the paper.

## Supplementary Material

Supplemental data

## Version 1 07/30/2020

In-Press Preview

## Version 2 09/03/2020

Electronic publication
