# Supplementary material for: ANGPTL8 has both endocrine and autocrine effects on substrate utilization
Source: JCI Insight. 2020 Sep 3;5(17):e138777. doi: 10.1172/jci.insight.138777 (PMC7526440; doi:10.1172/jci.insight.138777)

ANGPTL8 has Both Endocrine and Autocrine Effects on Substrate Utilization

Federico Oldoni[1*], Haili Cheng[1*,] Serena Banfi[1], Viktoria Gusarova[2], Jonathan C. Cohen[3], and Helen H. Hobbs[1,4]

[*]These authors contributed equally to this work. [1]Departments of Molecular Genetics and Internal Medicine, [2]Regeneron Pharmaceuticals, Tarrytown, NY 10591, [3]The Center for Human Nutrition, and [4]Howard Hughes Medical Institute, University of Texas Southwestern Medical Center, Dallas, TX 75390

The authors have declared that no conflict of interest exists.

Supplementary Tables

Table S1. Offspring of matings of different genotypes

**A. +/+ × Adipo-*Cre*Tg/+**								
	Males n=309 (51%)				Females n=298 (49%)			
	Observed		Expected		Observed		Expected	
Genotype	No.	%	No.	%	No.	%	No.	%
+/+	167	54%	154	50	146	49%	149	50
Tg/+	142	46%	155	50	152	51%	149	50
**B. +/+ × Alb-*Cre*Tg/+**								
	Males n=50 (52%)				Females n= 46 (48%)			
	Observed		Expected		Observed		Expected	
Genotype	No.	%	No.	%	No.	%	No.	%
+/+	25	50%	25	50	19	41%	23	50
Tg/+	25	50%	25	50	27	59%	23	50
**C. f/+ × f/+**								
	Males n=105 (47%)				Females N=119 (53%)			
	Observed		Expected		Observed		Expected	
Genotype	No.	%	No.	%	No.	%	No.	%
+/+	35	33%	24	25	33	26%	21	25
f/+	42	40%	57	50	59	48%	77	50
f/f	28	27%	24	25	27	26%	21	25
**D. f/f; +/+ x f/f; Adipo-*Cre*Tg/+**								
	Males n= 230 (50%)				Females n= 229 (50%)			
	Observed		Expected		Observed		Expected	
Genotype	No.	%	No.	%	No.	%	No.	%
(f/f)(+/+)	123	53%	115	50	108	47%	115	50
(f/f)(Tg/+)	107	47%	115	50	121	53%	114	50
**E. f/f; +/+ x f/f; Alb-*Cre*Tg/+**								
	Males n=191 (59%)				Females n= 134 (41%)			
	Observed		Expected		Observed		Expected	
Genotype	No.	%	No.	%	No.	%	No.	%
(f/f)(+/+)	91	48%	95	50	83	62%	67	50
(f/f)(Tg/+)	100	52%	96	50	51	38%	67	50

Table S2. Litter size of different genotypes

**A. +/+ × Adipo-CreTg/+**					
Breedings			Pups		
Male	Female	No. of litters	Pups per litter	Males	Females
+/+	Tg/+	45	4.8	108	108
Tg/+	+/+	85	4.9	216	201
**B. +/+ × Alb-CreTg/+**					
Breedings			Pups		
Male	Female	No. of litters	Pups per litter	Males	Females
+/+	Tg/+	7	5.6	21	18
Tg/+	+/+	11	7.54	41	42
**C. f/+ × f/+**					
Breedings			Pups		
Male	Female	No. of litters	Pups per litter	Males	Females
(f/f)	(f/f)	5	7.2	19	17
(f/+)	(f/+)	56	4.5	118	132
(+/+)	(+/+)	1	4.0	3	1
**D. f/f; +/+ x f/f; Adipo-CreTg/+**					
Breedings			Pups		
Male	Female	No. of litters	Pups per litter	Males	Females
(f/f)(+/+)	(f/f)(Tg/+)	62	4.7	140	154
(f/f)(Tg/+)	(f/f)(+/+)	34	3.9	60	73
**E. f/f; +/+ x f/f; Alb-CreTg/+**					
Breedings			Pups		
Male	Female	No. of litters	Pups per litter	Males	Females
(f/f)(+/+)	(f/f)(Tg/+)	38	4.0	93	57
(f/f)(Tg/+)	(f/f)(+/+)	32	5.5	93	83

Table S3. Oligonucleotides used for RT-PCR

Gene	Forward primer, 5′-3′	Reverse primer, 5′-3′
36B4	CACTGGTCTAGGACCCGAGAAG	GGTGCCTCTGGAGATTTTCG
ACC	TGGACAGACTGATCGCAGAGAAAG	TGGAGAGCCCCACACACA
ACL	GCCAGCGGGAGCACATC	CTTTGCAGGTGCCACTTCATC
ACS	GCTGCCGACGGGATCAG	TCCAGACACATTGAGCATGTCAT
ADIPOQ	AGATGGCACTCCTGGAGAGAA	TTCTCCAGGCTCTCCTTTCCT
ADRB2	GGTTATCGTCCTGGCCATCGTGTTTG	TGGTTCGTGAAGAAGTCACAGCAAGTCTC
ADRB3	TCTAGTTCCCAGCGGAGTTTTCATCG	CGCGCACCTTCATAGCCATCAAACC
AGPAT1	GCTGGCTGGCAGGAATCAT	GTCTGAGCCACCTCGGACAT
AGPAT2	TTTGAGGTCAGCGGACAGAA	AGGATGCTCTGGTGATTAGAGATGA
AGPAT3	CCAGTGGCTTCACAAGCTGTAC	CCCTGGGAATACACCCTTCTG
ANGPTL3	AGCAAGACAACAGCATAAGAGAACTC	TGAGCTGCTTTTCTATTTCTTTTATCTG
ANGPTL4	GCCTTTCCCTGCCCTTCTC	GATTGGAATGGCTACAGGTACCA
ANGPTL8	ACATGGCTGTGCTTGCTCTCT	CAAATTCTTGGTGGGCTTGAC
ATGL	GAGAGAACGTCATCATATCCCACTT	CCACAGTACACCGGGATAAATGT
CD36	GGAACTGTGGGCTCATTGC	CATGAGAATGCCTCCAAACAC
CHREBPα	CGACACTCACCCACCTCTTC	TTGTTCAGCCGGATCTTGTC
CHREBPβ	TCTGCAGATCGCGTGGAG	CTTGTCCCGGCATAGCAAC
CIDEA	CCGAGTACTGGGCGATACAGA	GGTTACATGAACCAGCCTTTGG
CPT1A	CACCAACGGGCTCATCTTCTA	CAAAATGACCTAGCCTTCTATCGAA
CPT2	AGCCTACCTGGTCAATGCATATC	GGGTTTGGGTATACGAGTTGAATT
CYCLO	TGGAGAGCACCAAGACAGACA	TGCCGGAGTCGACAATGAT
DIO2	CATTGATGAGGCTCACCCTTC	GGTTCCGGTGCTTCTTAACCT
ELOVL6	TGTACGCTGCCTTTATCTTTGG	GCGGCTTCCGAAGTTCAA
Eva1	CCACTTCTCCTGAGTTTACAGC	GCATTTTAACCGAACATCTGTCC
FABP4	ACTGGGCGTGGAATTCGATGA	ACCAGCTTGTCACCATCTCGT
FABP5	CGGGTCTATGAGAAGGTGCAA	GAGCATATTCACTCTGGCAGCTAA
FAS	GCTGCGGAAACTTCAGGAAAT	AGAGACGTGTCACTCCTGGACTT
GLUT4	CCGGCAGCCTCTGATCAT	CCGACTCGAAGATGCTGGTT
G6P	TGGGCAAAATGGCAAGGA	TCTGCCCCAGGAATCAAAAAT
HK2	TGCCAAGCGTCTCCATAAGG	GGAGGAAGCGGACATCACAA
HMGCR	CTTGTGGAATGCCTTGTGATTG	AGCCGAAGCAGCACATGAT
HPRT	CCTCATGGACTGATTATGGACAG	AATCCAGCAGGTCAGCAAAG
HSL	GGAGCACTACAAACGCAACGA	TCGGCCACCGGTAAAGAG
LPL	ACTCTGTGTCTAACTGCCACTTCAA	ATACATTCCCGTTACCGTCCAT
LXRA	TCTGGAGACGTCACGGAGGTA	CCCGGTTGTAACTGAAGTCCTT
PGC1α	AACCACACCCACAGGATCAGA	TCTTCGCTTTATTGCTCCATGA
PPARα	ACAAGGCCTCAGGGTACCA	GCCGAAAGAAGCCCTTACAG
PPARγ	CACAATGCCATCAGGTTTGG	GCTGGTCGATATCACTGGAGATC
PPARγ2	TGCCTATGAGCACTTCACAAGAAAT	CGAAGTTGGTGGGCCAGAA
PPARδ	ACGCACCCTTTGTCATCCA	TTCCACACCAGGCCCTTCT
PRDM16	CCCCACATTCCGCTGTGAT	CTCGCAATCCTTGCACTCA

RGS16	GGGCTCACCACATCTTTGAC	TTGGTCAGTTCTCGGGTCTC
SCD1	CCGGAGACCCCTTAGATCGA	TAGCCTGTAAAAGATTTCTGCAAACC
SREBP-1c	GGAGCCATGGATTGCACATT	GGCCCGGGAAGTCACTGT
SREBP-2	GCGTTCTGGAGACCATGGA	ACAAAGTTGCTCTGAAAACAAATCA
TXNIP	TATGTACGCCCCTGAGTTCCA	GTTAAGGACGCACGGATCCA
UCP1	ACTGCCACACCTCCAGTCATT	CTTTGCCTCACTCAGGATTGG

Supplementary Figures

Fig. S1

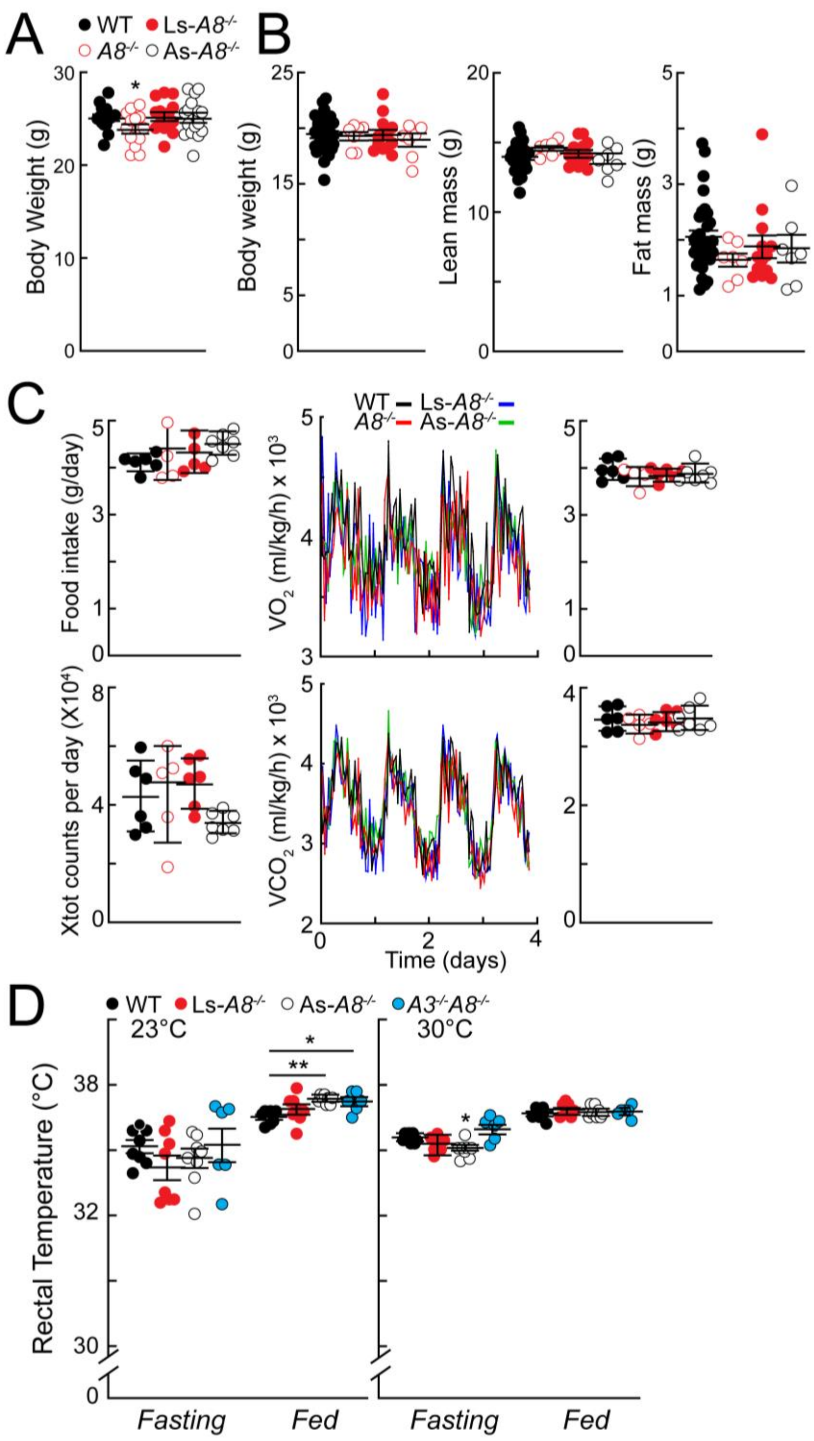

Figure S1

Body weights (A), indirect calorimetry (B) and rectal temperatures (C) of WT(fl/fl), *A8*$^{-/-}$, Ls-*A8*$^{-/-}$, and As-*A8*$^{-/-}$ male mice. (A) Body weights (± SEM) were obtained from age-matched male mice (n=4-5/genotype, 9-14 wk). Data shown were pooled from 3 independent experiments (14/genotype) and compared using two-way ANOVA. (B) Body weights (± SEM) of age-matched female mice (n=7-32/genotype, 9-10 wk). (C) Age-matched male mice (n=5–7/genotype, 11-16 wk) were housed individually in metabolic cages for seven days. Food intake and activity were monitored (right). Both O_2 consumption and CO_2 output were measured for 2 min at 50-min intervals for 4 consecutive days (right). Group means ± SEM for VO_2 consumption and VCO_2 output are provided (right panels). (D) Age-matched male Mice (n=6-8 per group, 12-15 wk) were housed individually in metabolic cages at 30°C for 3 weeks. Rectal temperatures were obtained at the end of a 15 h fast (left) and then 4 h after chow was provided (right). Groups were compared using one-way ANOVA with Dunnett`s multiple comparison test. * P<0.05, **P <0.01.

Fig. S2

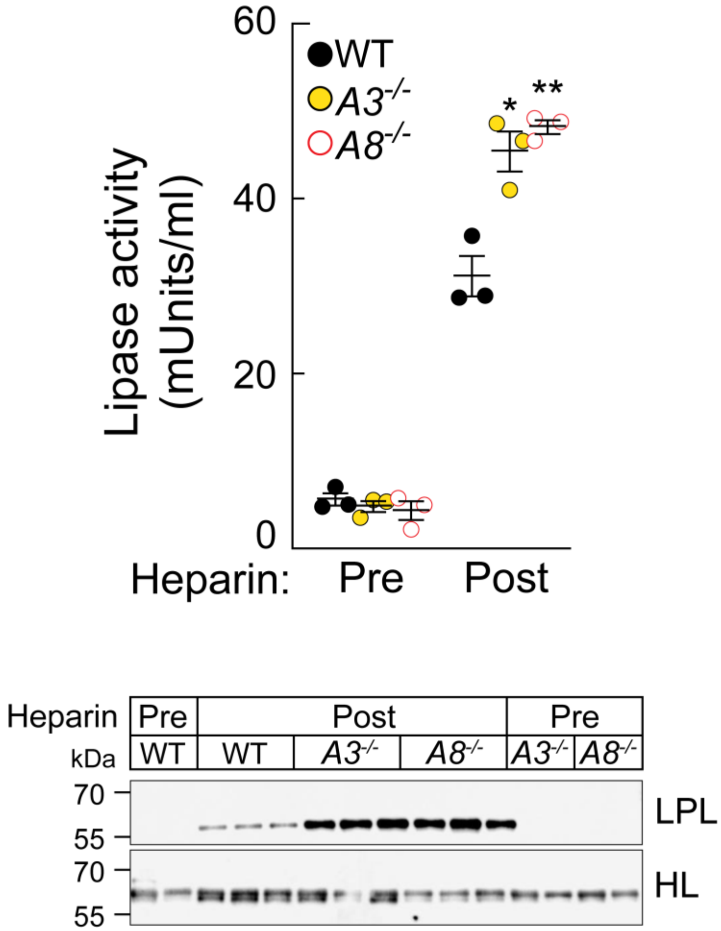

Figure S2

Intravascular LPL activity and mass are similar in *A3*$^{-/-}$ and *A8*$^{-/-}$ mice. The diets of the male mice (n=3/genotype, 13-16 wk) were synchronized for 3 days as described in the Methods. Four hours after refeeding, blood was obtained from WT mice and the mice were injected with heparin intravenously (1 U/g). Blood was collected after 15 min and lipase activity was

measured as described in the legend to Figure 5 (Top). A total of 10 μl of plasma from each mouse was diluted to 500 μl in PBS and incubated with 20 μl of heparin beads for 2 h. The heparin-bound proteins were subjected to immunoblotting for LPL and hepatic lipase (HL). Groups were compared using one-way ANOVA with Dunnett`s multiple comparison test. *P<0.05, **P <0.01.

Fig. S3

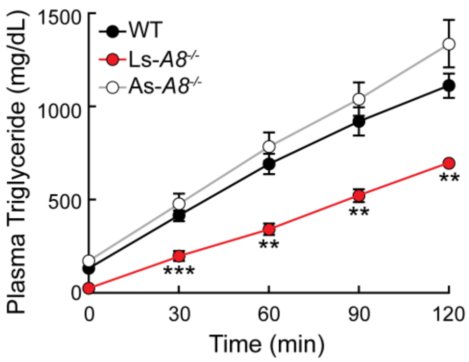

Figure S3.

VLDL-TG secretion rates in WT, Ls-*A8*$^{-/-}$ and As-*A8*$^{-/-}$ mice. The diets of female mice (n=4/genotype, 7-9 wk) were synchronized for 3 days as described in the Methods. At the end of the fasting cycle on day 3, mice were given access to food for 4 hours and then injected with a bolus of Triton WR-1339 (500 mg/kg) via the tail vein. Blood was collected from the tail vein at the indicated times, and TG levels were measured in plasma. The slopes of the lines were calculated assuming a linear increase in TG concentrations over time, and compared using unpaired two-tailed Student's *t* tests. **P<0.01, ***P<0.001.

Fig. S4

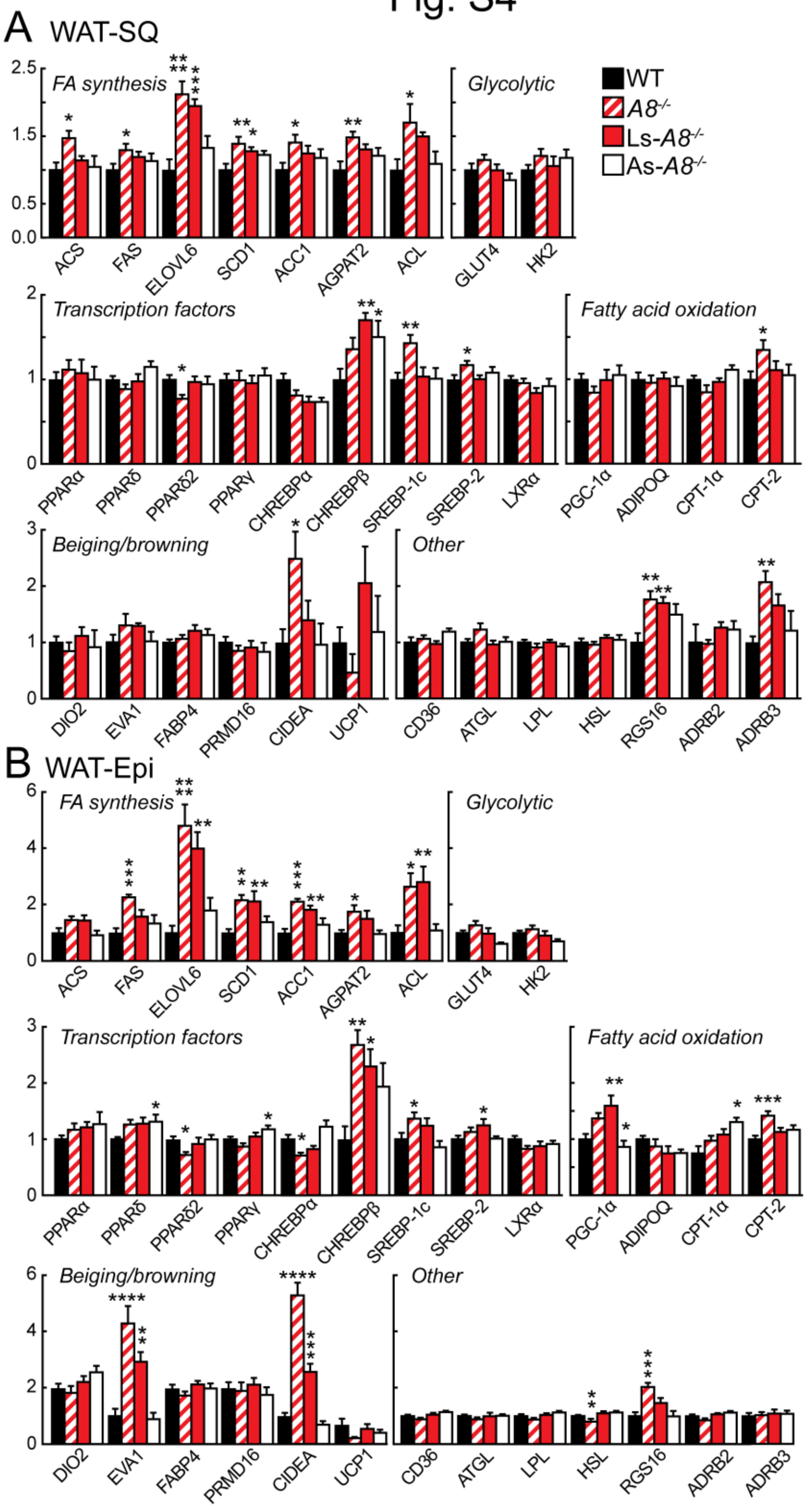
A WAT-SQ
FA synthesis
Glycolytic
WT
A8-/-
Ls-A8-/-
As-A8-/-
ACS
FAS
ELOVL6
SCD1
ACC1
AGPAT2
ACL
GLUT4
HK2
Transcription factors
Fatty acid oxidation
PPARα
PPARδ
PPARδ2
PPARγ
CHREBPα
CHREBPβ
SREBP-1c
SREBP-2
LXRα
PGC-1α
ADIPOQ
CPT-1α
CPT-2
Beiging/browning
Other
DIO2
EVA1
FABP4
PRMD16
CIDEA
UCP1
CD36
ATGL
LPL
HSL
RGS16
ADRB2
ADRB3
B WAT-Epi
FA synthesis
Glycolytic
Transcription factors
Fatty acid oxidation
Beiging/browning
Other

Fig. S4

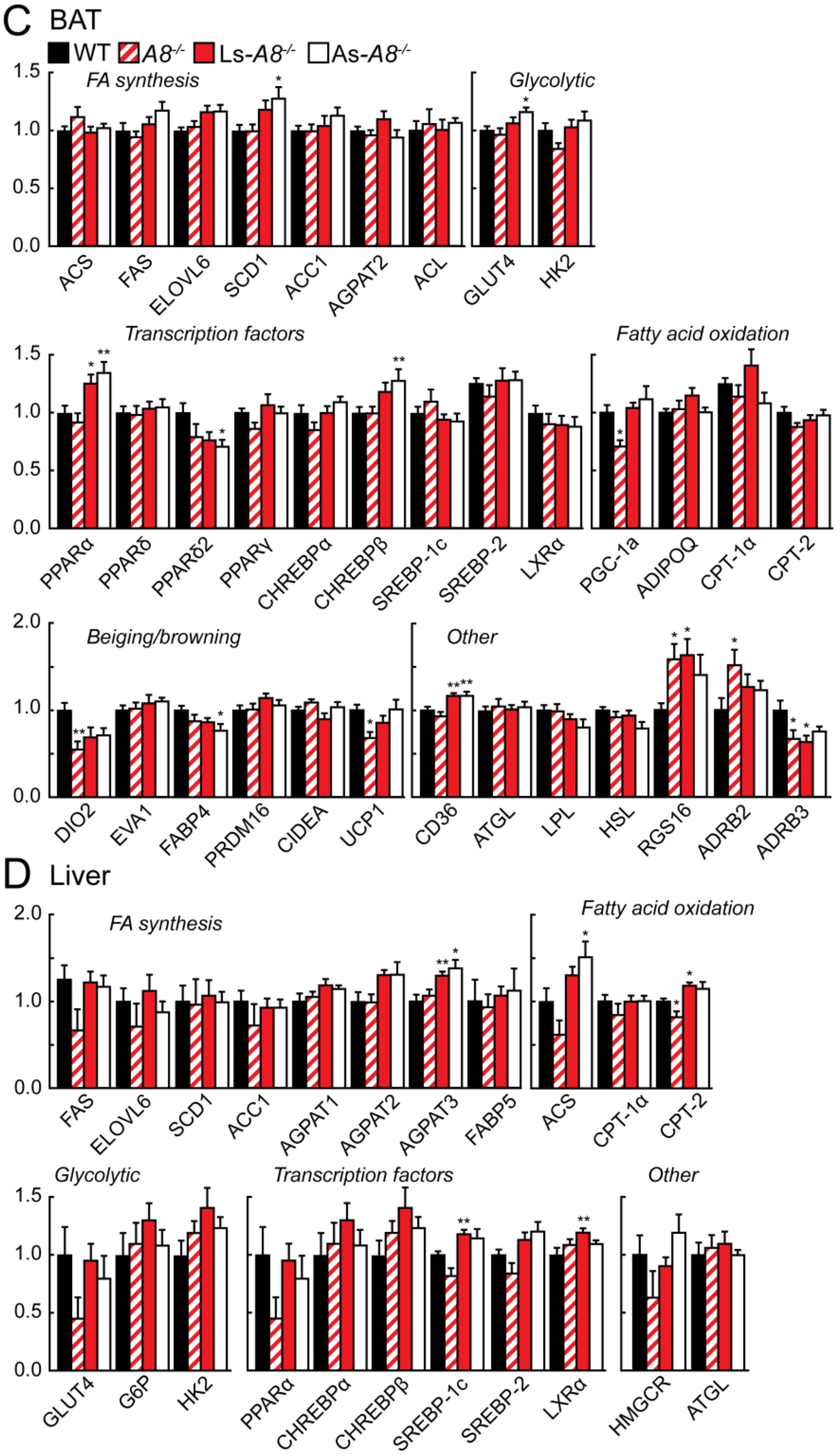

Figure S4.

Messenger RNA levels in adipose tissue and liver of male mice of the indicated genotypes. Male mice (n=6/genotype, 12-13 wk) were fasted overnight and then fed for 4 hours. Fold changes in levels of selected mRNAs encoding enzymes and transcription factors involved in fatty synthesis and oxidation, glycolysis, and thermogenesis/ browning are shown. Transcript levels were determined by Real-Time-PCR as described in the Methods. Data are expressed as means ± SEM. Groups were compared using one-way ANOVA with Dunnett`s multiple comparison test. *P < 0.05; **P < 0.01; ***P < 0.001, ****P < 0.0001. The experiment was repeated once, and the results were similar.

Fig. S5

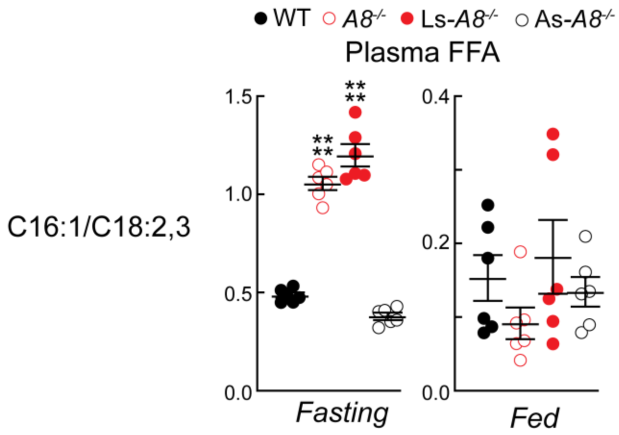

Figure S5.

C16:1 and C18:2,3 levels in circulating free fatty acids.

Levels of C16:1 (endogenously synthesized) and C18:2 and C18:3 (diet-derived) were measured in fasting and fed plasma free fatty acid pool as described in the Methods and the mean ratios (± SEM) of C16:1 to C18:2 plus C18:3 is shown. Groups were compared using one-way ANOVA with Dunnett`s multiple comparison test. ****P <0.0001.

Fig. S6

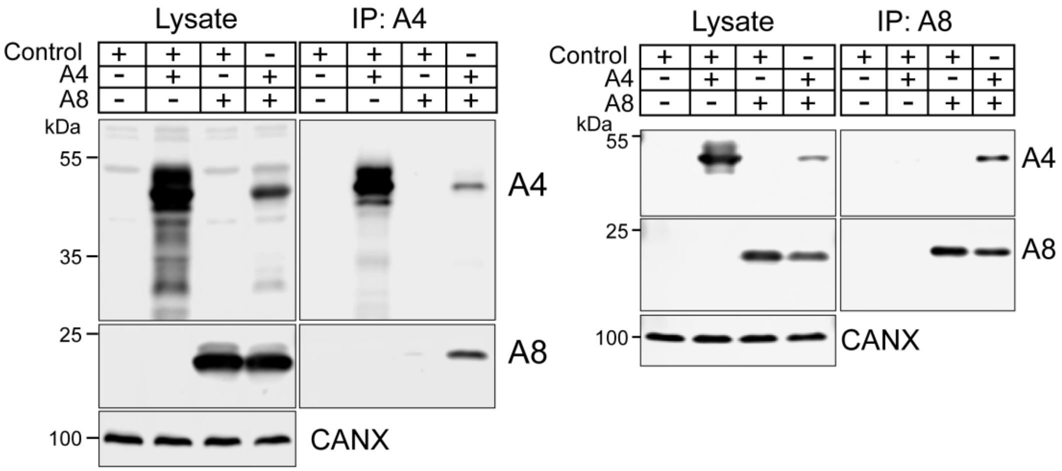

Figure S6

A8 and A4 physically interact in CHO-K1 cells. Recombinant A4-myc and A8-Flag were co-expressed in CHO-K1 cells alone (with empty plasmid) or together. A4 was immunoprecipitation using Myc-linked magnetic beads (left panel) and A8 was immunoprecipitated using Flag-magnetic beads (right panel) as described in the Methods. As A4 has non-specific binding to magnetic beads, Flag-beads were eluted by 3X Flag peptide. Immunoblot analysis using anti-A4 (top), anti-A8 (middle) and anti-calnexin (CANX) Abs was performed using 30 μg of lysate (input) as described in Methods.

Full unedited gel for Figure 3A

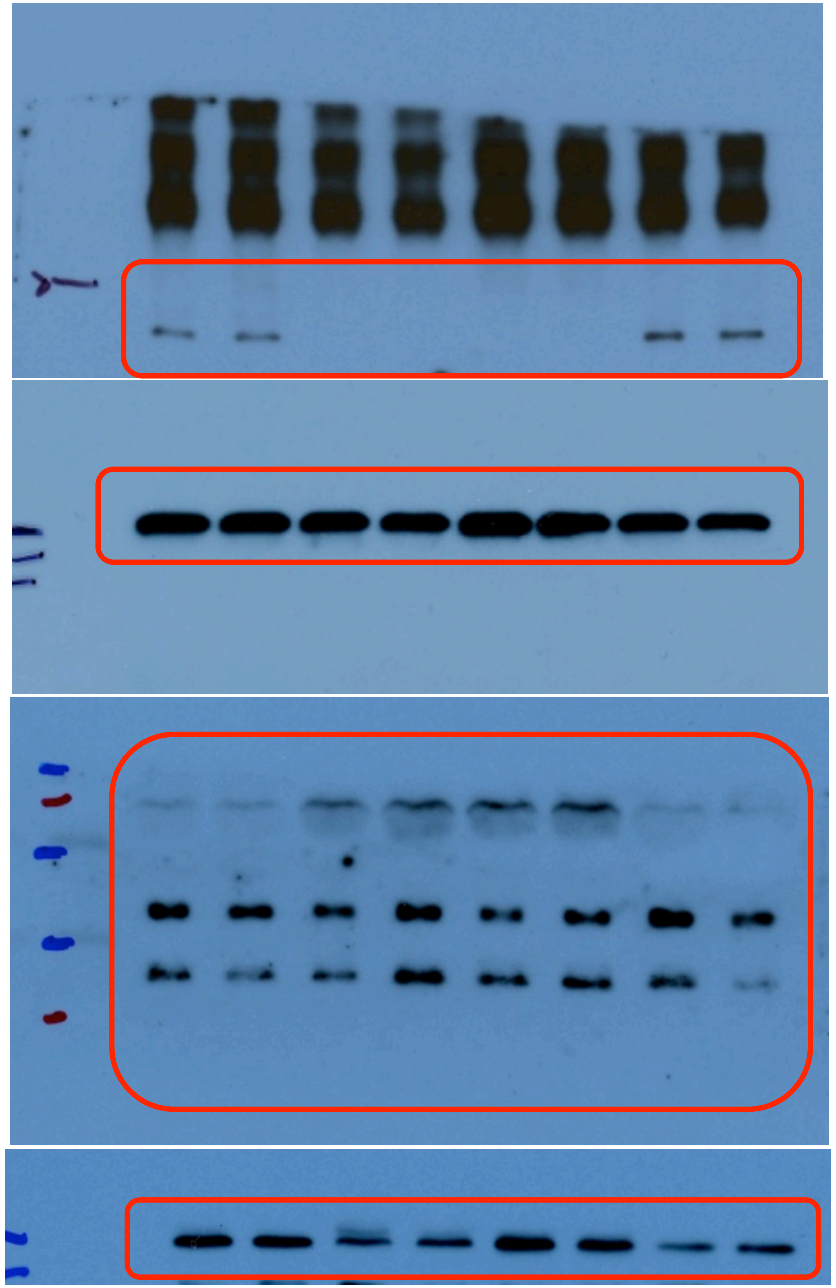

Full unedited gel for Figure 3B

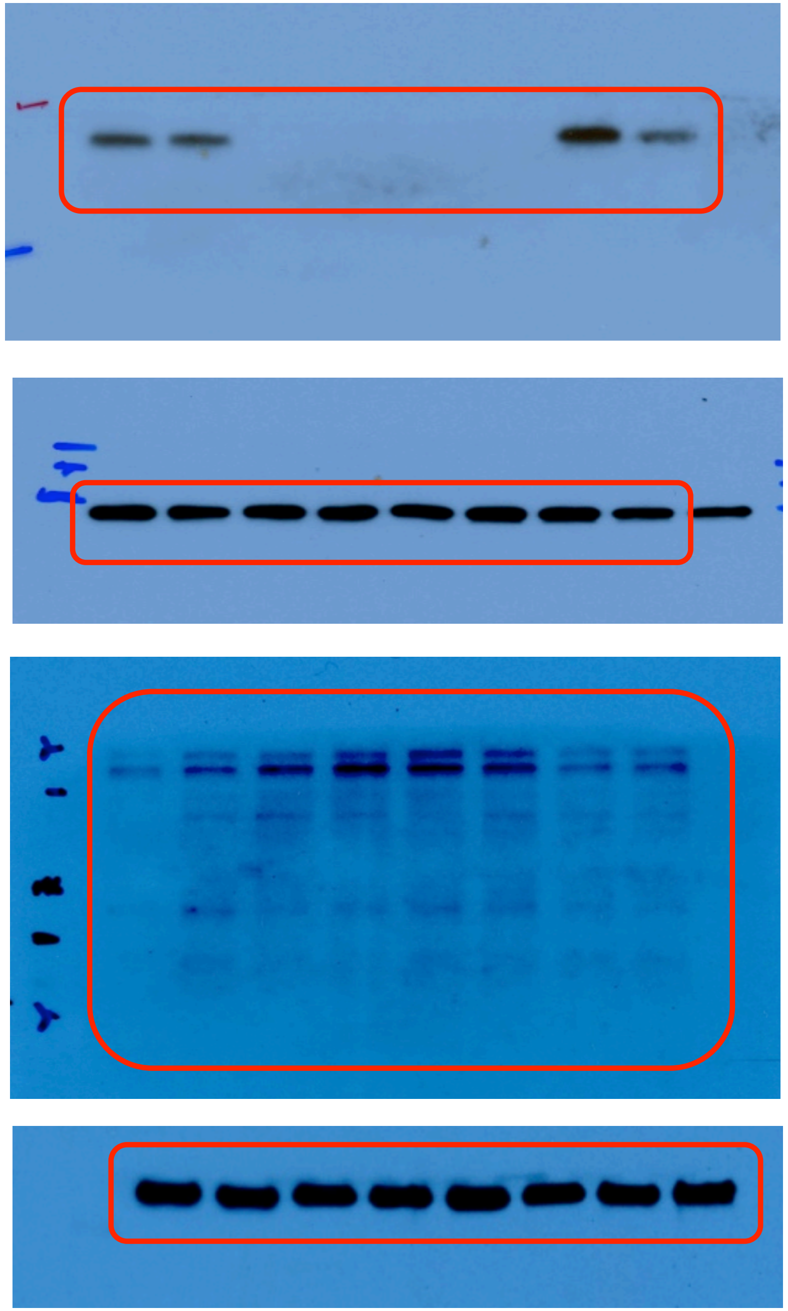

Full unedited gel for Figure 4A

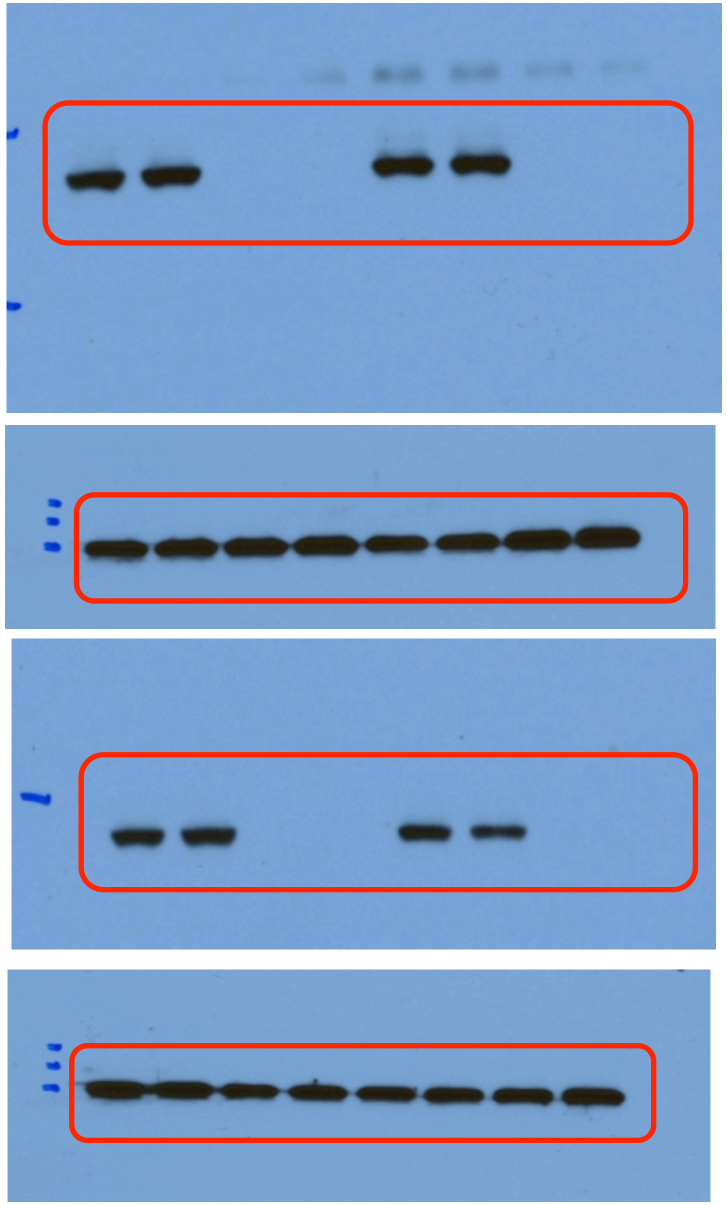

Full unedited gel for Figure 7C

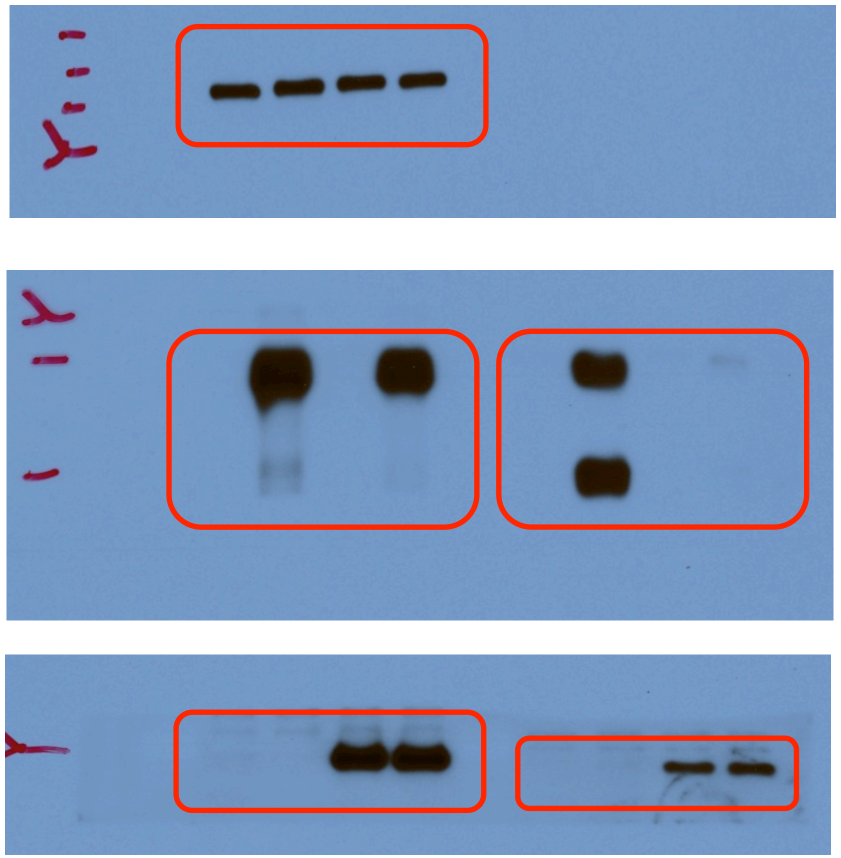

Full unedited gel for Figure 5C

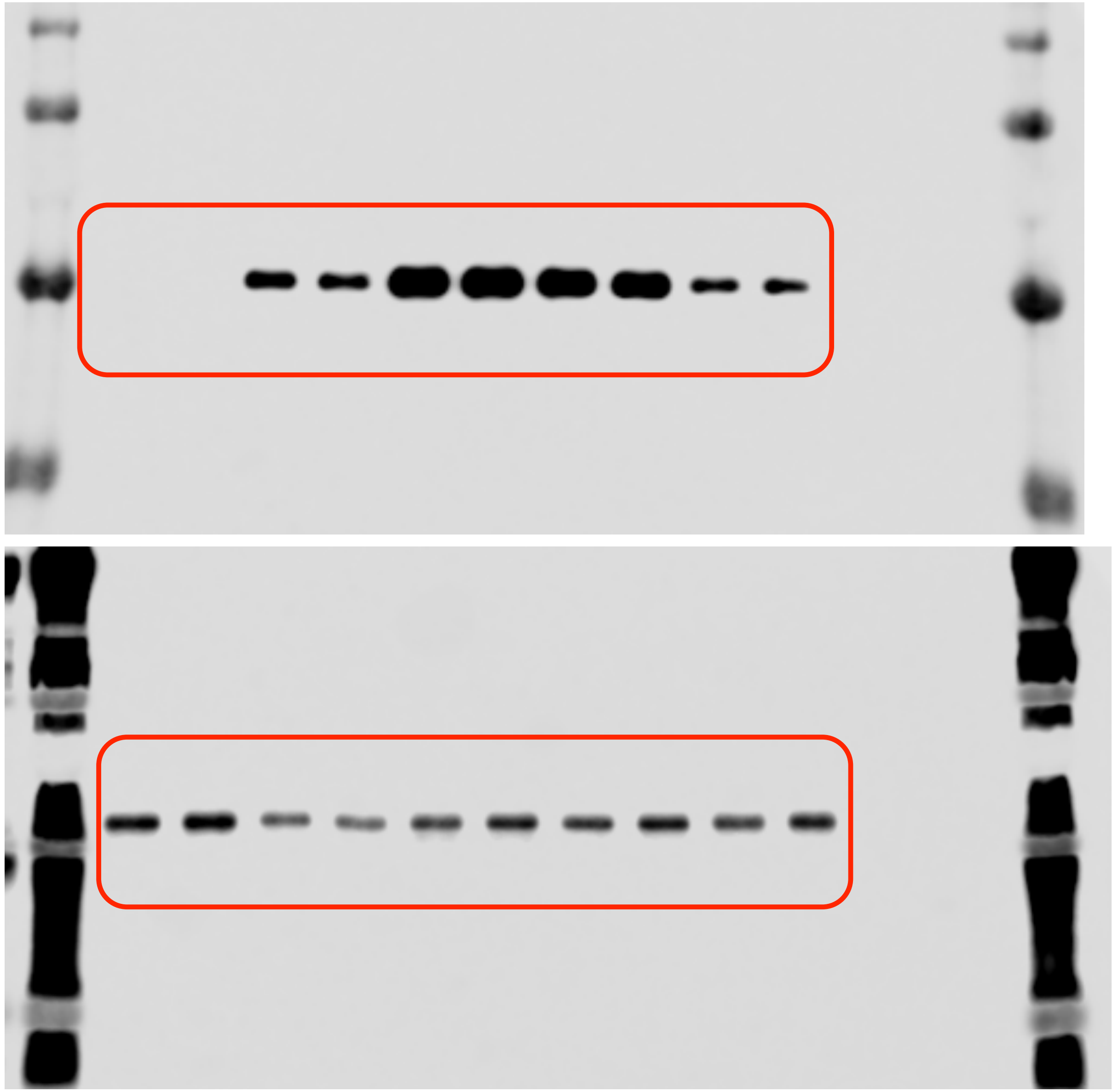

Full unedited gel for Figure 7A

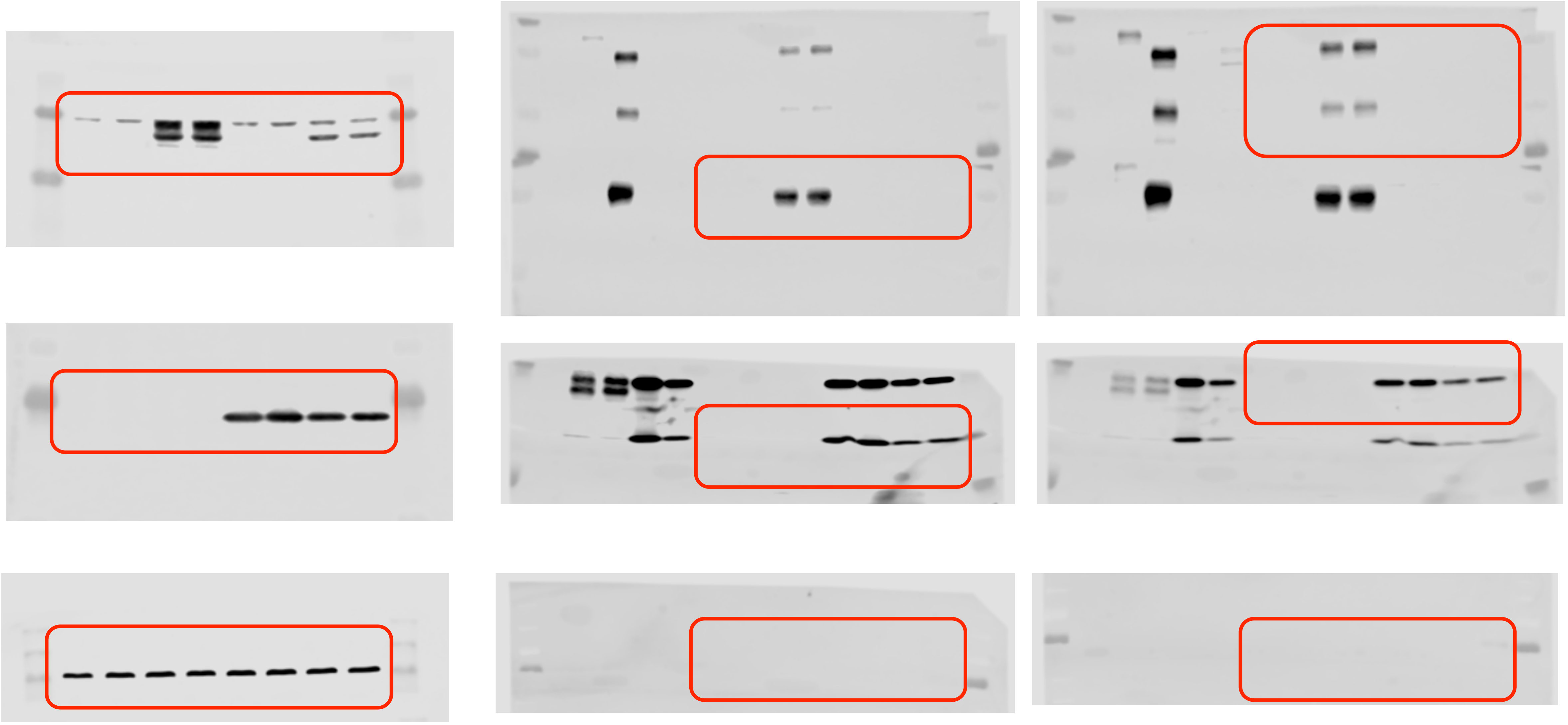

Full unedited gel for Figure 8A

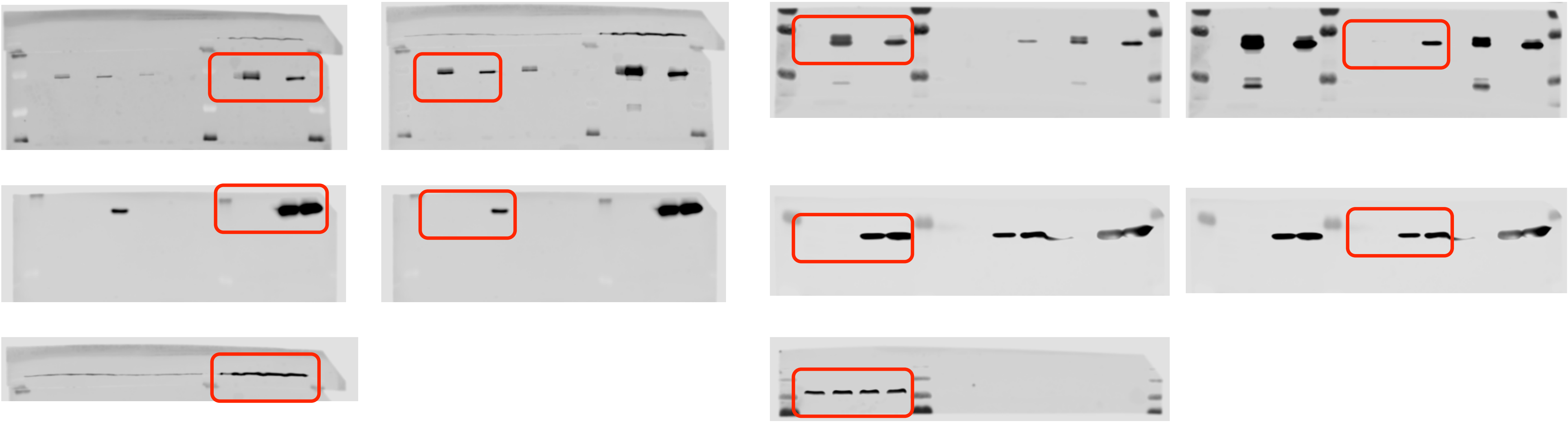

Full unedited gel for Figure S2

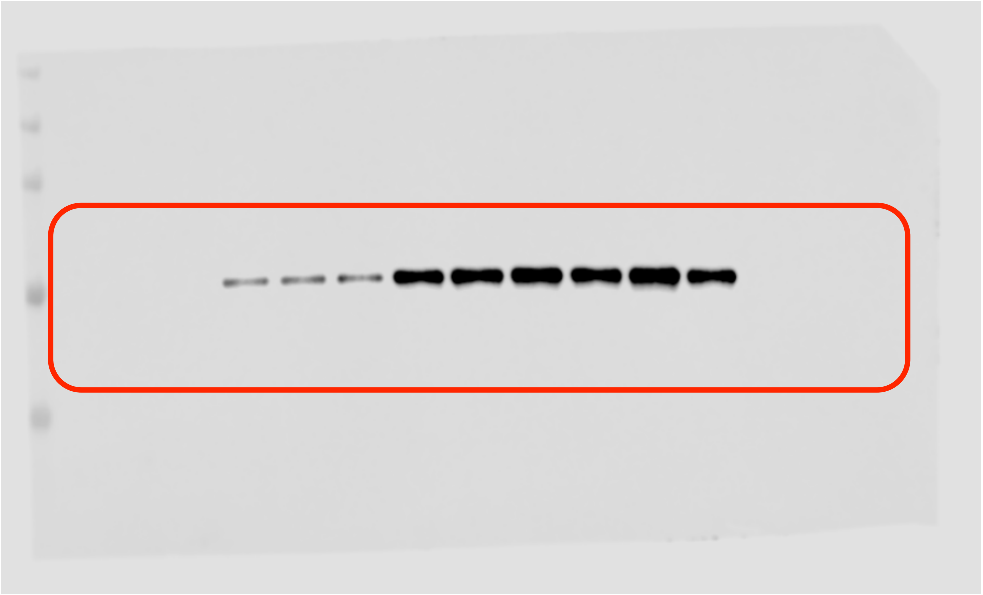

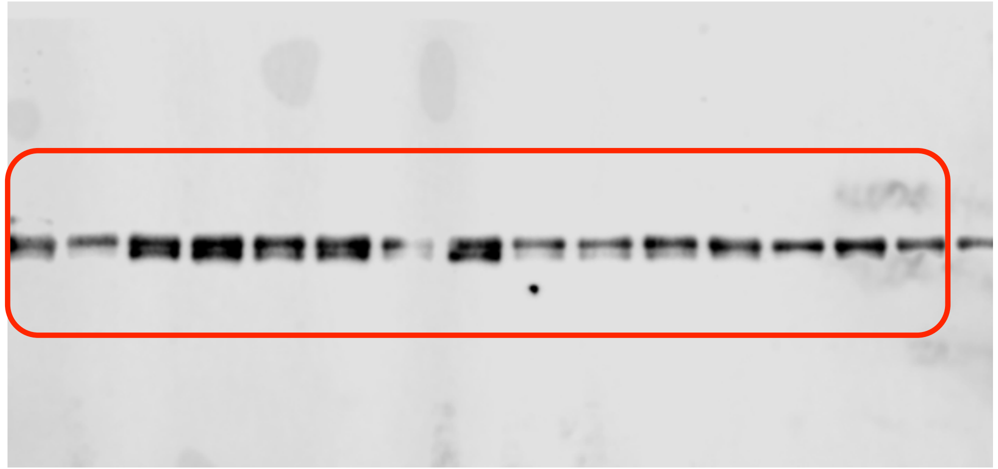

Full unedited gel for Figure S6

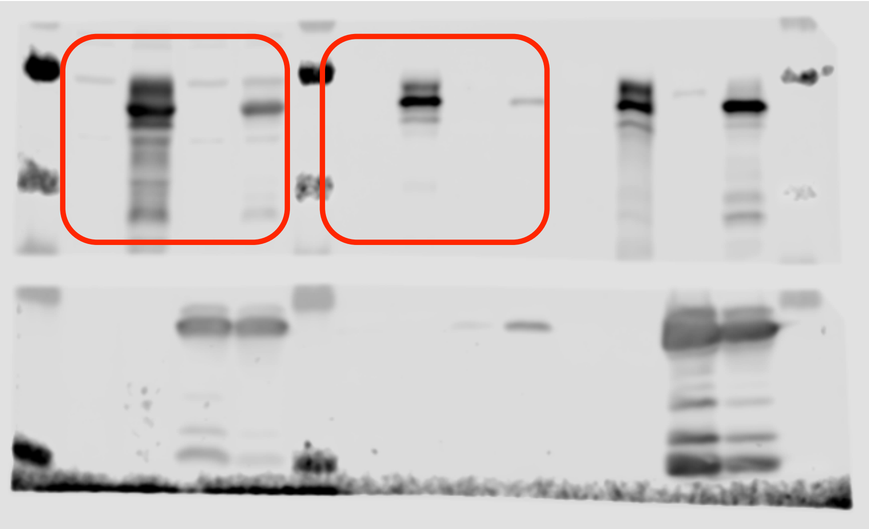

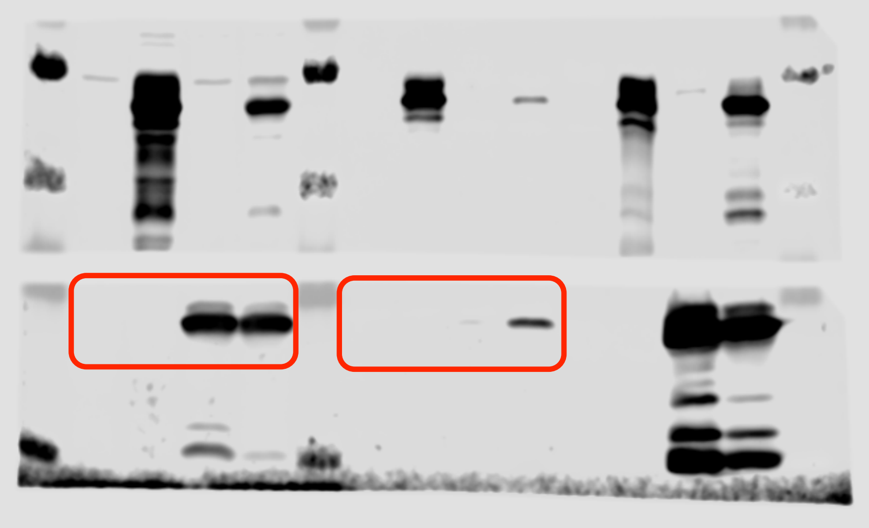

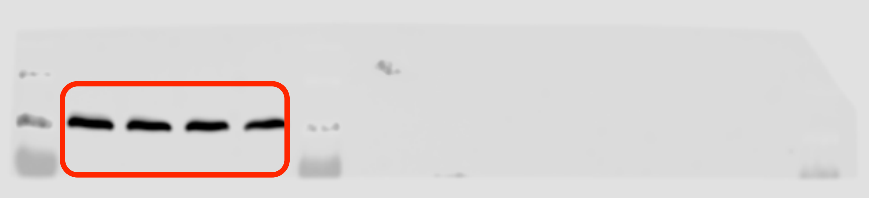

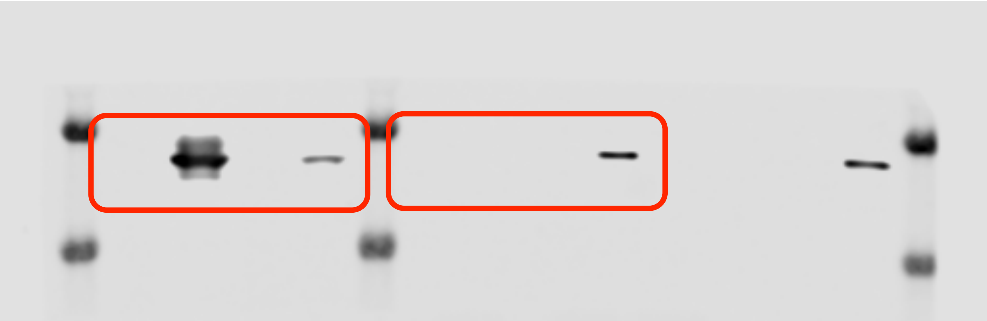

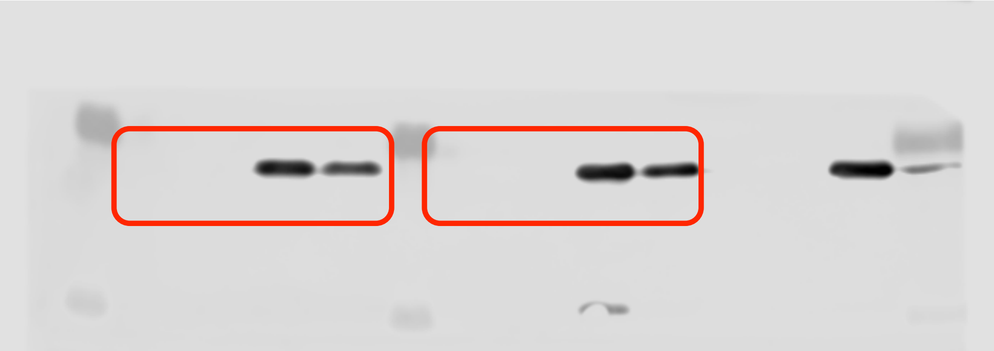

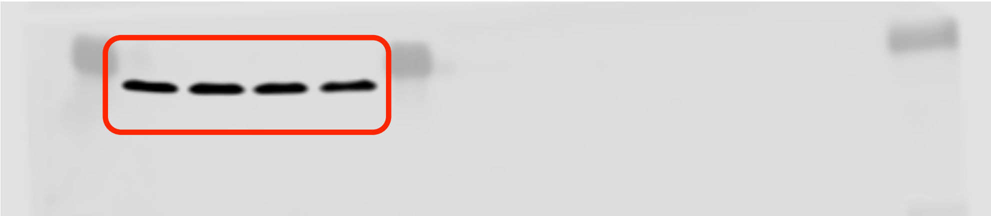

Supplement: Supplemental data [file jciinsight-5-138777-s033.pdf]
